# An Exploratory LC-HRMS Metabolomics Study of Culture Medium-Dependent Metabolic Variation and Bioactivity in Ten Fungal Strains

**DOI:** 10.3390/ijms27093866

**Published:** 2026-04-27

**Authors:** Ria Desai, Gagan Preet, Rishi V. Astakala, Adriana Romero-Otero, Pilar Sanchez, Thomas A. Mackenzie, Thomas O. Larsen, Rainer Ebel, Marcel Jaspars

**Affiliations:** 1 Marine Biodiscovery Centre, Department of Chemistry, University of Aberdeen, Aberdeen AB24 3UE, UK; ria.desai@abdn.ac.uk (R.D.); gagan.preet1@abdn.ac.uk (G.P.); rishi.astakala1@abdn.ac.uk (R.V.A.); r.ebel@abdn.ac.uk (R.E.); 2 Department of Biotechnology and Biomedicine, Technical University of Denmark, 2800 Kongens Lyngby, Denmark; aromero@dtu.dk (A.R.-O.); tol@bio.dtu.dk (T.O.L.); 3 Fundación MEDINA, Avda Conocimiento 34, 18016 Granada, Spain; pilar.sanchez@medinaandalucia.es (P.S.); thomas.mackenzie@medinaandalucia.es (T.A.M.)

**Keywords:** fungal secondary metabolism, untargeted metabolomics, OPLS regression, metabolite-bioactivity association, cytotoxic activity, molecular networking

## Abstract

Fungi represent a prolific source of structurally diverse secondary metabolites, yet the extent to which culture conditions reshape the metabolic profile and functional bioactivity remains incompletely understood. In this exploratory study, ten fungal strains belonging to genera *Penicillium* and *Aspergillus* were cultivated in Yeast Extract Sucrose (YES) and Czapek Yeast Autolysate (CYA) media and analysed using untargeted LC-HRMS metabolomics. The objective of this study was to evaluate how culture medium influences metabolic profiles and to investigate medium-dependent metabolic variation and its relation to cytotoxic, antibacterial, and antifungal activities. Global metabolic profiling revealed moderate but statistically significant medium-associated metabolite variation, with discriminant metabolites predominantly enriched under CYA conditions. Putative structural annotation suggested patterns consistent with differential regulation of isoprenoid-derived sterols, terpenoids, alkaloid-like metabolites, and aromatic polyketides. While antimicrobial activities displayed a heterogeneous, strain-dependent pattern with limited correlation to individual metabolites, cytotoxic activity co-varied with metabolite composition in OPLS regression modelling. Sterols and terpenoid-related features emerged as major contributors to cytotoxicity. Given the absence of biological replication and the limited sample size inherent to this pilot study, all findings should be considered hypothesis-generating and interpreted within an exploratory framework. These results suggest that nutrient composition influences biosynthetic pathway activation while functional outcomes remain strongly dependent on strain-specific metabolic capacity. This work provides a systematic framework and targeted hypothesis for future investigations into condition-dependent fungal chemical diversity in natural product discovery.

## 1. Introduction

Fungi are among the richest sources of structurally diverse metabolites, many of which have led to clinically relevant compounds with antimicrobial, anticancer, and immunomodulatory effects. Despite the characterisation of over 30,000 fungal secondary metabolites to date, genomic analyses consistently indicate that this number represents only a fraction of their true biosynthetic potential [1]. The majority of these biosynthetic gene clusters remain silent or poorly expressed under standard laboratory conditions, highlighting the importance of cultivation strategies to explore chemical diversity. Recent reports indicate that uncovering chemo-diversity in fungal species is now increasing with trends showing 553 novel fungal metabolites reported in 2023 [2] and 907 metabolites in 2024 [3]. This included polyketides, terpenoids, and alkaloids as dominant structural classes. However, studies show that even for well-studied *Aspergillus* and *Penicillium* genera, 80–90% of metabolites remain uncharacterised, and 84% of biosynthetic gene clusters (BGCs) are understudied, suggesting that their metabolic capacity remains underexplored [1,2,4]. Importantly, the challenge is not only the discovery of new metabolites, but also the systematic identification of culture conditions that activate distinct pathways and integrating them into functional bioactivity [5].

Secondary metabolism in fungi is highly responsive to environmental and nutritional changes. In laboratory conditions, variations in medium composition, carbon and nitrogen sources, and cultivation parameters can also strongly influence metabolite production. This concept underlies the ‘One Strain Many Compounds’—OSMAC—approach, which exploits changes in culture conditions to induce a diverse chemical profile and might activate otherwise silent biosynthetic pathways from a single strain. Though medium variation alters metabolite abundance, the extent to which these changes translate to measurable bioactivity output remains poorly understood.

Untargeted high-resolution mass spectrometry (HRMS)-based metabolomics provides a powerful approach to address this gap. By combining comprehensive feature detection, multivariate statistical modelling, and molecular networking, metabolomics enables systematic comparisons of fungal extracts across culture conditions. Utilising integrative statistical approaches like PCA, OPLS-DA, and regression modelling allows the discrimination of medium-dependent metabolic variation and facilitates the identification of metabolites associated with functional outcomes like cytotoxicity or antimicrobial activity.

In this exploratory study, untargeted LC-HRMS metabolomics and multivariate statistical analyses were applied to ten fungal extracts in two commonly used media, YES and CYA. As each strain–medium combination was cultivated as a single replicate, this study is exploratory in design, and all statistical analyses are intended to identify patterns and prioritise candidate metabolites for future hypothesis-driven work. Our objectives were to (i) evaluate the influence of culture medium and species identity on metabolic profile, (ii) identify discriminating metabolite families associated with medium-dependent variation, and (iii) investigate the relationship between metabolic composition and measured cytotoxic, antibacterial, and antifungal activities. By integrating metabolomics-guided investigation, this work aims to provide a systematic framework for understanding how cultivation conditions, specifically different media, shape the fungal secondary metabolism and how these changes link to functional bioactivity in the context of natural product discovery.

## 2. Results and Discussion

### 2.1. Retention Time Distribution of Metabolic Features Between Culture Media

Fungal strains were grown on two common culture media, Yeast Extract Sucrose (YES) and Czapek Yeast Autolysate (CYA), to evaluate the influence of media composition on metabolic output. YES is a nutrient-rich medium containing readily available simple sugars and amino acids, whereas CYA provides a more complex carbon source and a defined nutrient balance. These contrasting nutrient environments are known to modulate secondary metabolism in fungal culture.

**Figure 1:**
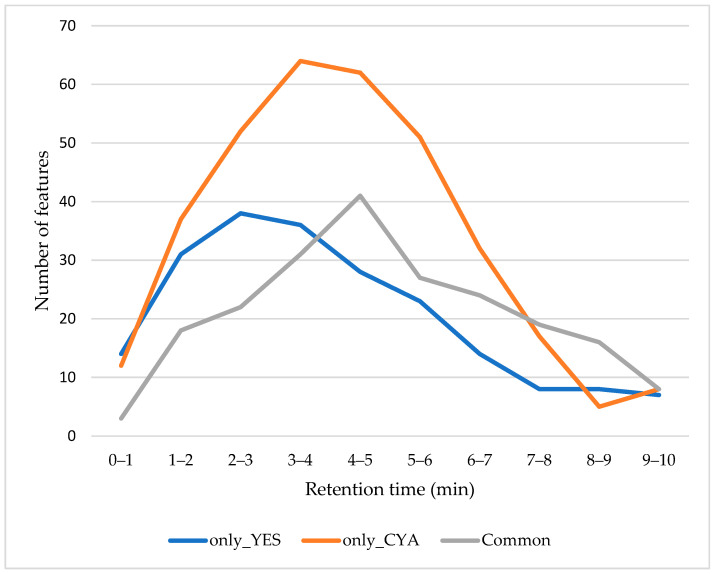
Retention time distribution of LC-MS features by medium condition. Each bar represents the count of features unique to YES extract (blue), unique to CYA extract (yellow), and shared between both media (grey) in each group.

Untargeted LC-HRMS detected approximately 756 metabolic features across all extracts after removing signals present in medium control and quality control samples. To examine the distribution of chemical diversity across chromatographic space and to compare medium-dependent differences, detected features were grouped into 1 min retention time (RT) intervals. They were further classified as (i) unique to YES, (ii) unique to CYA, or (iii) shared between both media. As shown in Figure 1, the retention time distribution of features varies markedly across two cultural media. CYA extracts consistently exhibited a higher number of unique features across most RT intervals, particularly within a 3–6 min elution window. This region typically corresponds to metabolites of intermediate polarity, which is consistent with the common polarity range of many fungal secondary metabolites, suggesting broader induction of metabolic pathways under CYA cultivation. In contrast, YES extracts displayed features across a full chromatographic range but at lower counts per interval compared to CYA. Features detected in both media were present throughout the retention time range, indicating a shared core metabolome, though there were fewer than the CYA-specific features across the start to mid-late retention interval (0–7 min).

Notably, CYA-specific features extended into mid-late retention time regions, suggesting the presence of less polar or structurally complex metabolites as in reverse phase chromatography. These patterns may reflect the enhanced production of media-responsive secondary metabolites under CYA cultivation. YES-specific features were represented by the relatively early to mid-retention time interval associated with polar metabolites. Although retention time alone does not confirm compound polarity, these trends suggest differential regulation of metabolite classes in response to nutrient composition. Overall, the RT analysis suggests that culture medium not only influences the number of features but also their chromatographic distribution, indicating shifts in the chemical profile of fungal metabolome.

### 2.2. Multivariate Analysis of Metabolic Profile

**Figure 2:**
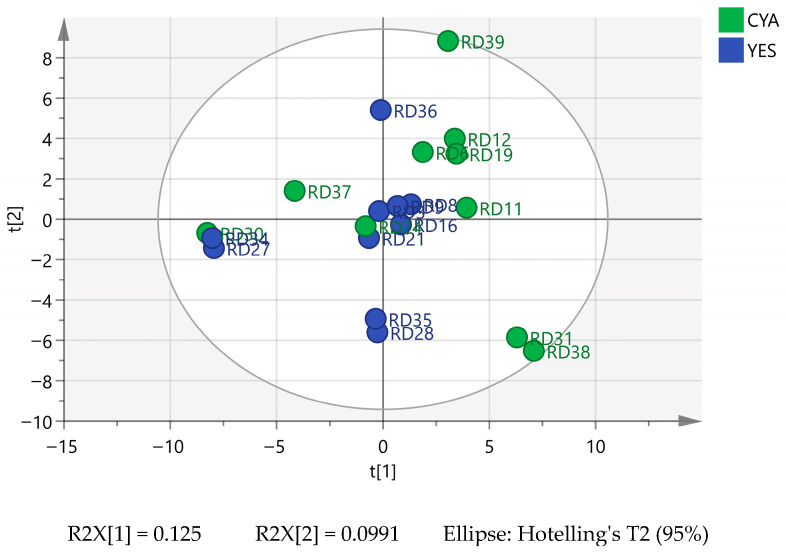
PCA score plot of 10 fungal metabolic profiles grown on medium CYA and YES. Samples are coloured by cultivation medium, with green for CYA and blue for YES.

Unsupervised principal component analysis (PCA) was performed on a normalised LC-MS dataset to assess global metabolomic variation among fungal extracts in two culture conditions (YES vs. CYA). The first two principal components explained 12.5% (PC1) and 9.9% (PC2) of total variance, capturing ~22.4% of overall metabolic variation (Figure 2).

Partial separation between YES and CYA extracts was observed along PC1, indicating medium-associated differences in the global metabolite profile. This variation was statistically supported by permutation multivariate analysis of variance (PERMANOVA), which revealed a significant medium effect (F = 4.35, R^2^ = 0.07, *p* = 0.017). However, a relatively low R^2^ value indicates that the culture medium accounts for a quite small proportion (~7%) of total metabolic variance. Such a pattern is common in untargeted LC-MS datasets, where hundreds of features contribute to high-dimensional variation distributed across multiple principal components [6,7]. For example, in *Penicillium roqueforti* populations, the first two principal components accounted for only ~33% of total variance, with substantial variation distributed across later components [8]. In the complex fungal metabolome, variance often reflects the overlapping influence of medium composition, as also observed in this study, along with strain-level biosynthetic capacity and shared core metabolic pathways rather than a single dominant driver [9,10,11].

Species identity did not form clearly distinct clusters within the PCA space. Extracts belonging to the same species did not consistently group, suggesting that taxonomic identity was not the primary driver of principal axes variation captured by PC1 and PC2, since both media accounted for 7% of metabolic variance. The remaining variance likely reflects the overlapping contribution of shared fungal core metabolome and strain-level biosynthetic differences. Overall, samples were interspersed, indicating substantial shared chemical space across taxa. However, certain strains displayed more distant positioning in the score plot, like how *Penicillium expansum* (RD31, RD38) and *Penicillium scabrosum* (RD39) grown on CYA appeared separated from the centre cluster. These deviations likely reflect a strain-specific metabolic profile or a differential response to medium conditions rather than analytical outliers. Similar observations have been reported in fungal metabolomics studies where individual strains exhibit different metabolic profiles within an overall medium influence framework [12]. Within *Penicillium* and *Aspergillus*, the genera studied here, individual isolates from different ecological origins frequently exhibit divergent secondary metabolic profiles even within the same species, with strain identity contributing substantially to metabolic variation [13]. Mycotoxin production and cytotoxicity studies across *Penicillium*, *Aspergillus*, and *Alternaria* isolates from diverse environmental sources similarly showed substantial strain-level variation in metabolite profile, toxigenicity, and cytotoxic properties [14]. In these studies, culture medium composition was also reported to influence metabolic patterns, with substantial strain-dependent variation in cytotoxicity. This is further supported by a study of 23 actinomycete isolates, which identified six PCA outliers with a unique metabolic profile that yielded previously unreported compounds, supporting the view that positional deviation reflects genuine metabolic distinctness rather than an analytical artefact [15]. Overall, the multivariate analysis indicates that culture medium exerts a statistically detectable but moderate influence on the fungal metabolome studied here. Substantial variation arises from intrinsic biological differences and shared metabolite production across strains.

Supervised multivariate methods (PLS-DA/OPLS-DA) were explored to enhance separation, but these models did not yield robust predictive performance, consistent with the moderate overlap observed in PCA scores and the limited sample size. This outcome aligns with previous reports demonstrating medium effects as detectable but not overwhelmingly strong relative to strain-specific metabolic variability. For example, studies on *Fusarium verticillioides* have demonstrated that different media selectively favour the production of particular mycotoxin profiles, without achieving complete separation of the overall metabolome [16]. These observations support the interpretation that medium effects are often pathway- or compound-specific rather than uniformly altering the global metabolomic profile. Accordingly, the supervised models described in the following sections are used for variable selection and pattern exploration rather than as confirmatory classifiers.

### 2.3. Media Discriminant Metabolites

To identify specific metabolites contributing to medium-associated differences, an orthogonal partial least squares discriminant analysis (OPLS-DA) model was constructed with medium as the class variable. Although the overall predictive performance of the model was moderate, the OPLS-DA framework remained useful for identifying variables contributing to medium-associated variation, particularly when combined with univariate statistical filtering. Features contributing to class separation were selected based on a variable importance in projection (VIP) threshold of more than 1.0.

**Figure 3:**
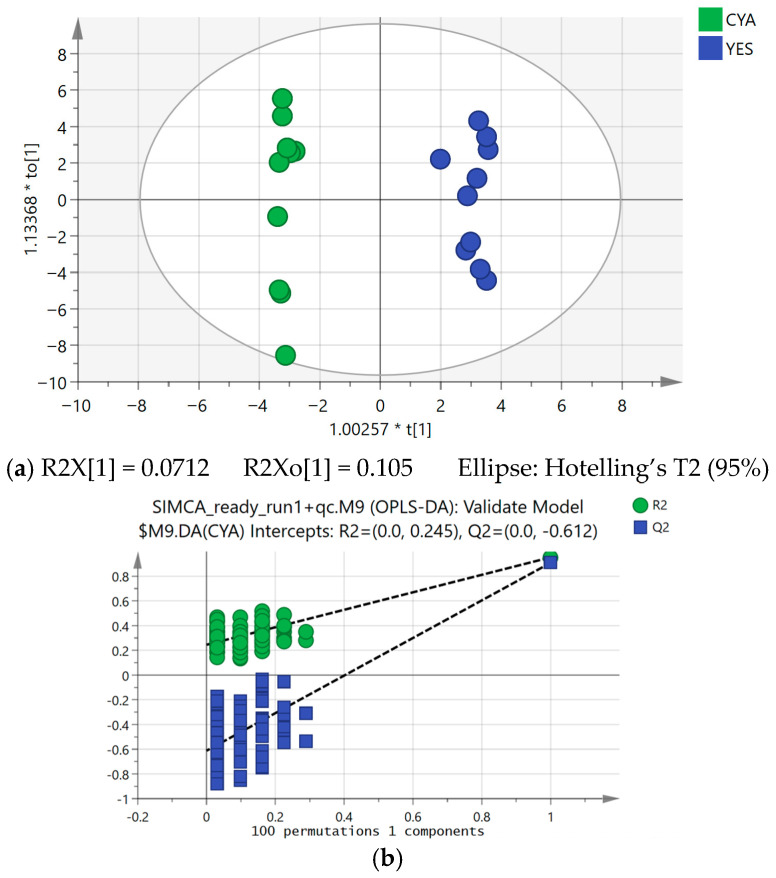
(**a**) Supervised OPLS-DA score plot (axis labels reflect SIMCA 14.1 display scaling conventions, where the coefficient preceding the asterisk denotes a normalisation scalar applied to component scores for visualisation purposes); (**b**) permutation testing (100 permutations).

Model robustness was confirmed by permutation testing (100 permutations; R^2^ intercept = 0.245 and Q^2^ intercept = −0.612) and cross-validation ANOVA (CV-ANOVA; F = 91.24 and *p* <0.05), indicating statistically significant class discrimination and absence of model overfitting (Figure 3 and Table 1). It is noted that the R^2^ intercept of 0.245, while acceptable, reflects residual structure in the permuted data, likely due to the small sample size rather than genuine overfitting, and is interpreted cautiously. To further refine the discriminant variables and incorporate univariate statistical validation, a volcano plot was generated using a *p*-value threshold of <0.05 and a fold change (FC) ≥ 1.5. Combining both multivariate (VIP) and univariate criteria identified 26 features that were significantly altered between media.

**Figure 4:**
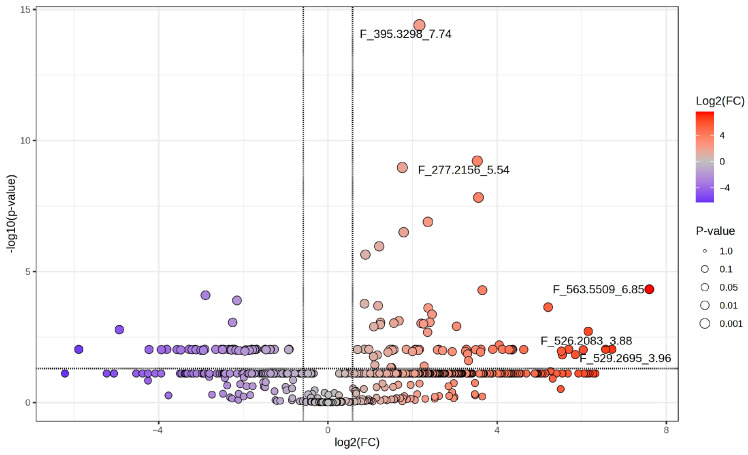
Volcano plot showing differential altered metabolites between CYA and YES extracts. Parameters selected *p*-value < 0.05 RAW and FC ≥ 1.5.

**Figure 5:**
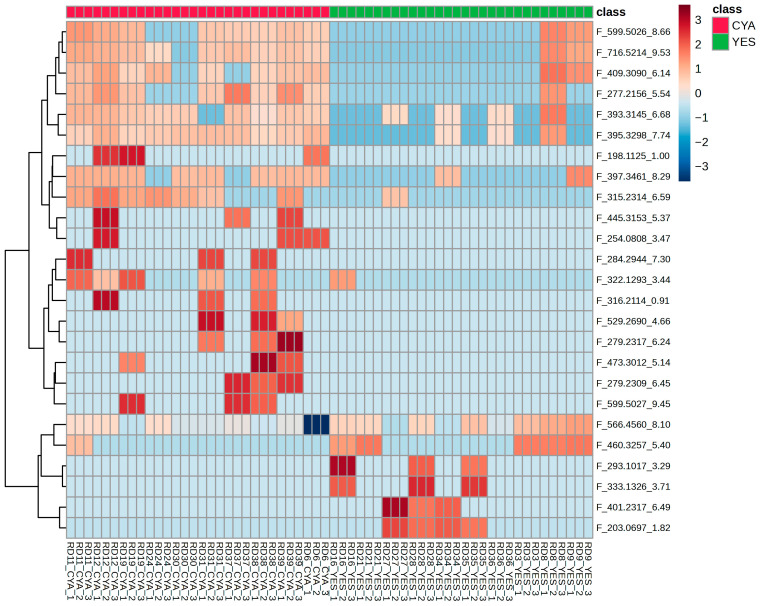
Hierarchical clustering heatmap of relative intensities of the top 25 significantly altered features across YES and CYA extracts.

Volcano plot analysis revealed striking asymmetry in feature distribution, with the majority of significant altered features enriched in the CYA medium compared to the YES medium, as shown in Figure 4. Only a limited subset of features was upregulated in YES medium. Hierarchical clustering of selected features demonstrated clear separation of samples according to culture medium, with tight clustering of replicate samples, supporting analytical reproducibility (Figure 5). Notably, most discriminant features exhibited higher relative abundance in CYA extracts, consistent with the OPLS-DA model.

#### 2.3.1. Putative Structural Annotations of Discriminant Metabolites

The 26 discriminant features were subjected to putative structural annotations using GNPS molecular networking and SIRIUS with CSI:FingerID in silico prediction. Annotations allowed for classification into major biosynthetic families (Table S1). Putatively annotated features included structure consistent with Roquefortine C, a griseofulvin-related metabolite, Fiscalin E analogue, and Tryhistatin-like compounds.

Several of these alkaloid-derived metabolites have been previously reported to exhibit antimicrobial activity and cytotoxic and neuroactive properties in fungal cultures. For example, Fiscaline E derivatives have demonstrated cytotoxic and antimicrobial activities [17,18,19] while Roquefortine C is a well-characterised indole alkaloid with reported cytotoxic and neurotoxic properties [20,21,22]. However, structural annotations are putative in the present study at confidence levels 2–3 based on spectral similarity and in silico fragmentation pattern and require further confirmation. All annotation levels follow Schymanski et al. [23]. Also, the literature review presented here cannot be directly attributed to these features without further validation. Their enrichment within medium-specific subnetworks suggests possible activation of biosynthetic pathways associated with nitrogen-containing secondary metabolites during CYA cultivation and represents a hypothesis for targeted follow-up investigation.

#### 2.3.2. Chemical Class Distribution of Discriminant Metabolites

**Figure 6:**
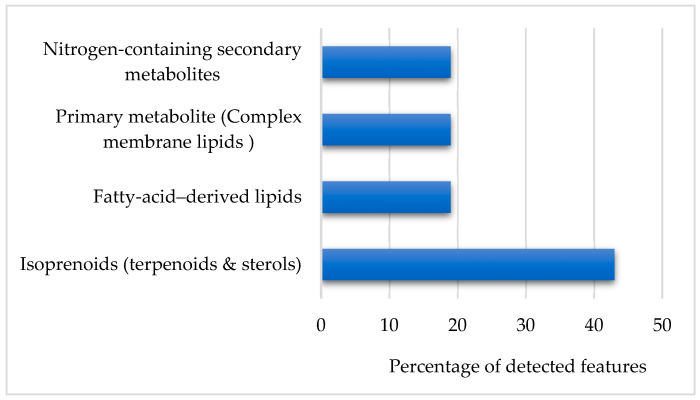
Bar graph showing broad chemical class distribution of top discriminant features.

Classification of discriminant metabolites revealed that isoprenoid-derived compounds represented the largest proportion of altered features, followed by fatty acid-derived lipids and nitrogen-containing secondary metabolites (Figure 6). The enrichment of sterol and lipid-related metabolites in CYA extracts suggests that this medium preferentially promotes pathways associated with membrane-associated secondary metabolism and terpenoid biosynthesis. Such pathway regulation is consistent with known nutrient-dependent control of fungal secondary metabolism, in which carbon and nitrogen availability modulate biosynthetic gene cluster expression through global regulatory networks [24,25].

#### 2.3.3. Molecular Networking

**Figure 7:**
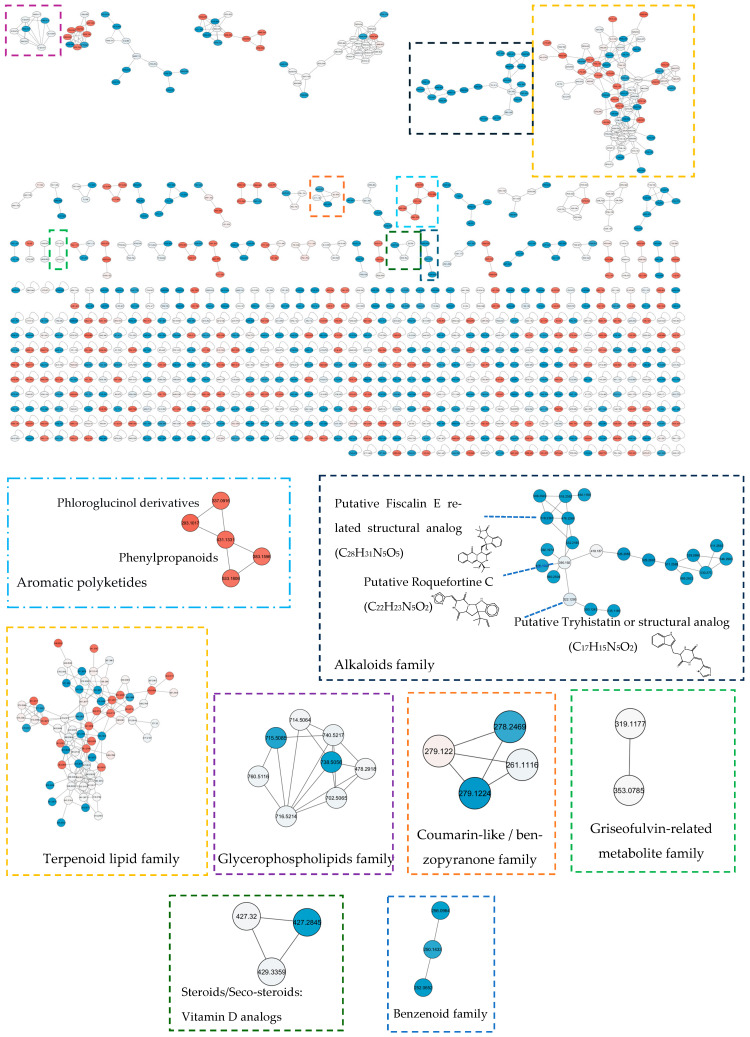
GNPS molecular network of fungal metabolome. Nodes represent MS/MS features; edges indicate spectral similarity (cosine score threshold applied). Node colour reflects log_2_ fold change (YES vs. CYA), with red indicating YES enrichment and blue indicating CYA enrichment. Major annotated clusters corresponding to steroidal, terpenoid, coumarin-like, lipids, terpenoids, and alkaloid clusters are highlighted.

The overall metabolic profile of 10 fungal extracts was organised into a GNPS molecular network, revealing diverse structural metabolite families (Figure 7). Major clusters correspond to terpenoid-related metabolites, alkaloid-like compounds, coumarin-type compounds, glycerophospholipids, and benzenoid derivatives. The feature annotation analyses were supported by SIRIUS and GNPS-based structural analysis, with all assignments representing putative identifications at confidence levels 2–3.

A prominent CYA-enriched cluster comprised nitrogen containing secondary metabolites, including features putatively consistent with Fiscalin E or analogues, Roquefortine C or related compounds, and Tryhistatin-like structures. Several coumarin-like and benzenoid metabolites also displayed CYA enrichment, suggesting coordinated activation of aromatic and alkaloid biosynthetic pathways under CYA cultivation.

In the YES-enriched cluster, along with terpenoid and lipid families, a cluster representing aromatic polyketide-related metabolites was observed. These metabolites are putatively identified as phloroglucinol-type scaffolds with neighbouring phenylpropanoid-like structures (coumarins/chromones). The antifungal metabolite griseofulvin [26] and its related analogue formed a small cluster with relatively comparable abundance in both media.

The differential metabolic outputs between YES and CYA are consistent with their contrasting nutritional composition. YES provides abundant, readily available carbon (150 g/L sucrose) and complex organic nitrogen (20 g/L yeast extract), conditions generally associated with the activation of primary growth pathways and carbon catabolite repression (CCR). This can suppress the expression of secondary metabolite BGCs [27]. In contrast, CYA represents a more defined and comparatively nutrient-limited environment, with lower complex nitrogen (5 g/L yeast autolysate) and inorganic nitrate as the primary nitrogen source. Nitrate assimilation requires additional metabolic regulation, associated with the relief of nitrogen metabolite repression, promoting secondary metabolism [28]. These conditions are also linked with reduced activity of global growth-regulating pathways such as TOR signalling, thereby decreasing secondary metabolite production in fungi [29]. The preferential enrichment of isoprenoid-derived sterols and terpenoids under CYA conditions is consistent with increased flux of resources towards secondary biosynthesis pathways, including the mevalonate pathway [24,30]. Similarly, enrichment of nitrogen-containing alkaloids reflects depression of alkaloid biosynthesis under conditions where readily available nitrogen is limited. In addition, the higher osmolarity of the YES medium, due to elevated sucrose concentration, may further favour primary metabolism over secondary metabolite production [28,31]. These observations support a model wherein nutrient-rich conditions favour primary growth, whereas a more balanced environment promotes secondary metabolite biosynthesis. While these mechanisms are consistent with established regulatory frameworks in fungal metabolism, direct validation would require transcriptomic or genomic analysis.

Collectively, these findings indicate that nutrient composition influences coordinated biosynthetic pathways rather than affecting individual metabolites. The structural clustering observed in molecular networking supports class-level distribution derived from in silico annotation. This also reinforces that medium composition reshapes the global fungal metabolome in a pathway-specific manner.

### 2.4. Association Between Metabolic Profiles and Bioactivity

#### 2.4.1. Bioactivity Pattern Across Media

Cytotoxic, antibacterial, and antifungal activity were evaluated across all the fungal crude extracts to determine whether medium-induced metabolic shifts correspond to measurable differences in functional bioactivity. Because antibacterial and antifungal assays involved multiple test organisms, Z-score normalisation was applied to these datasets to enable standardised comparison across strains (Table S2).

**Figure 8:**
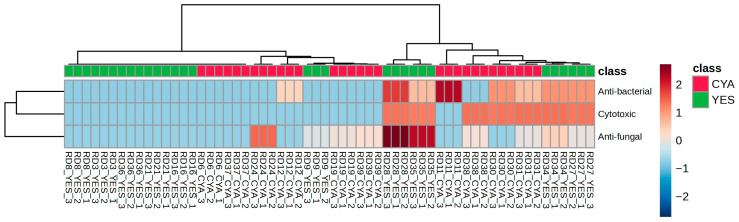
Bioactivity heatmap across fungal strains and media. Z-score normalised bioactivity profiles (cytotoxic, antifungal, antibacterial) for all extracts.

Hierarchical clustering of bioactivity profiles shown in Figure 8 revealed that activity patterns were predominantly strain-dependent rather than strictly medium-dependent. While cultivation medium influenced the intensity of response, species-specific inclination was more distinct.

Elevated cytotoxicity (Z > 1) was observed in both YES and CYA-derived extracts, indicating that cytotoxic potential was not completely associated with one medium. *P. expansum* strains from two distinct ecological origins (RD28/RD31 and RD35/RD38) exhibited comparable activity in both media, suggesting an intrinsic cytotoxic potential of the strain independent of culture media. Similarly, one *Aspergillus insuetus* strain pair (RD27/RD30) demonstrated activity under both media conditions. In contrast, the second *A. insuetus* pair (RD34/RD37) showed activity in only YES medium, despite duplicate experimental validation, indicating a genuine medium–strain interaction rather than technical variability.

These observations indicate that cytotoxic potential reflects strain-specific metabolic response to nutrient conditions and not a uniform media-driven effect, though this hypothesis requires confirmation in replicated studies.

Antifungal responses showed clear enhancement under YES conditions among strains exhibiting measurable activity. Notably, *P. expansum* strains (RD28/RD35) cultivated on YES medium showed higher antifungal activity compared to their CYA counterparts (RD31/RD38). A similar pattern was observed for *A. insuetus* strains, where YES-derived extracts (RD27/RD34) displayed antifungal activity whereas CYA extracts (RD30/RD37) showed minimal to no activity. Although *P. velutinum* cultivated on CYA medium (RD24) exhibited moderate antifungal activity, the overall profile favoured YES medium for the induction of antifungal metabolites in responsive species. These findings tentatively suggest that YES medium may preferentially stimulate biosynthetic pathways associated with antifungal secondary metabolites in selected strains. One possible explanation is that yeast-derived components present in YES extract including cell wall constituents such as β-glucans and mannoproteins may act as biological signals triggering a competitive antifungal response in the producing strains, analogous to interspecies chemical signalling mechanisms documented in fungal co-culture systems [32,33]. This hypothesis remains speculative in the present study and requires direct experimental testing, for example, through fractionation of yeast extract components or co-culture experimental designs.

In contrast, antibacterial activity was more heterogeneous and did not exhibit a consistent medium-associated pattern. Elevated antibacterial responses were observed in both YES and CYA extracts, depending on species. For *A. insuetus*, moderate activity was detected in both YES and CYA extracts, depending on ecological origin, indicating strain-level variability. Notably, *Penicillium hordei* cultivated in CYA medium (RD11) displayed strong antibacterial activity, while its YES culture (RD8) showed no activity. Similarly, *Penicillium freii* (RD12/RD9) demonstrated moderate activity with variation between media. This again suggests that antibacterial effects are likely governed by strain-specific biosynthetic capacity rather than a uniform nutrient-driven condition.

Overall, bioactivity clustering did not strictly follow medium identity alone but reflected an interaction between culture medium and species-specific metabolic potential. While medium clearly influenced the metabolic production as demonstrated in earlier multivariate analyses, the direction and intensity of its functional effects varied across strains. Interestingly, *P. expansum* and *A. insuetus* emerged as consistent bioactive species across multiple assays, suggesting a robust secondary metabolite reservoir capable of responding to environmental nutrient stimuli. These findings align with the established model of fungal metabolism, in which the nutrient environment shapes the secondary metabolism by acting as a regulatory trigger, influencing the activation and expression of biosynthetic pathways. However, the specific profile and abundance of bioactive compounds produced, which determine its phenotypic bioactivity, depend on the strain’s intrinsic genetic and biosynthetic potential [34]. Such medium–strain interactions are well documented in fungal metabolomics, where nutrient composition modulates the secondary metabolite gene cluster activation while species identity determines the available biosynthetic repertoire [24,35,36]. Thus, the bioactivity outcome in this dataset appears to reflect medium-influenced expression of strain-specific biosynthetic pathways rather than the uniform induction of bioactive compounds across all strains, a hypothesis that requires validation through follow-up studies with biological replication.

#### 2.4.2. Relation Between Medium Discriminant Features and Bioactivity

**Figure 9:**
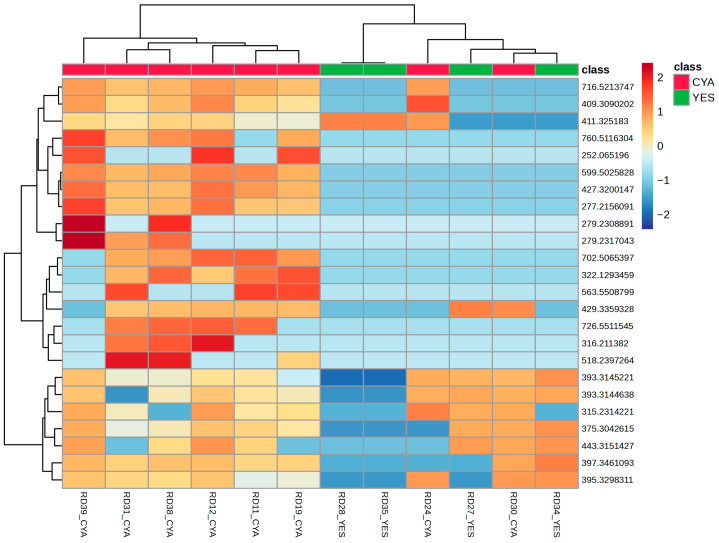
Hierarchical clustering heatmap showing the relative abundance of top media-discriminant metabolites in strains exhibiting measurable bioactivity.

Hierarchical clustering was performed using 26 selected features, restricted to strains exhibiting measurable cytotoxic or antimicrobial activity (Figure 9). This allowed for the evaluation of discriminant metabolites co-localising with bioactive phenotypes across strains.

To determine whether the metabolites discriminating YES and CYA media extracts were linked to measured bioactivity, Spearman correlation analysis was performed between the 26 identified discriminant features and the measured cytotoxic, antibacterial, and antifungal responses. Although most discriminant features were enriched in CYA media, the bioactivity profile did not uniformly favour CYA extracts. Therefore, correlation analysis was conducted without assuming directional media dependence.

**Figure 10:**
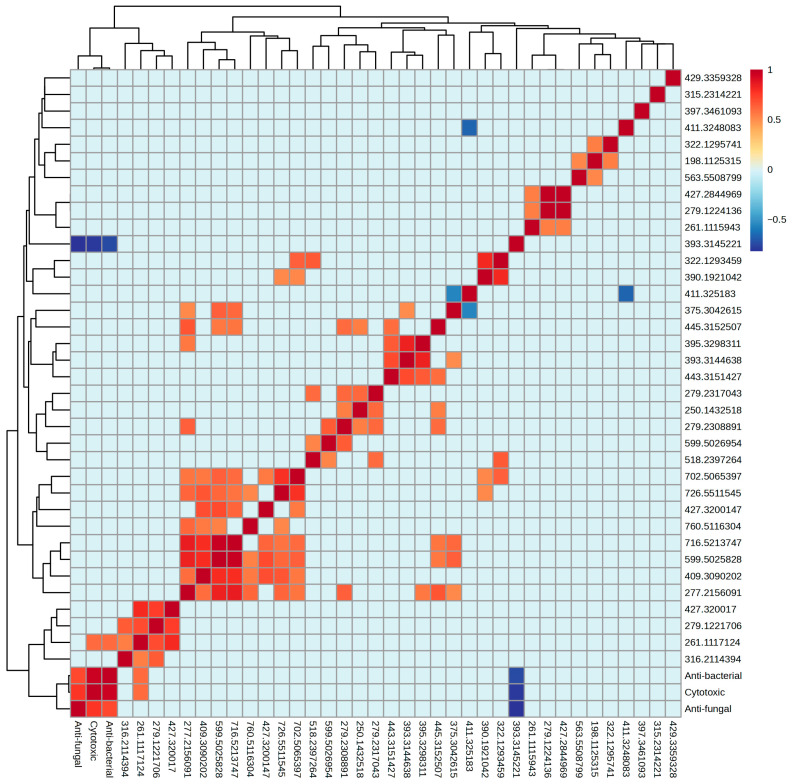
Spearman correlation heatmap between selected metabolites and bioactivity. Positive and negative correlations are indicated by colour gradients.

Overall, correlation coefficients between identified metabolites and antibacterial and antifungal activity were predominantly weak (Figure 10). No single metabolite demonstrated a strong, consistent positive association across antimicrobial activity. These findings suggest that antimicrobial bioactivity in this dataset is unlikely to be driven by single or few dominant metabolites but may reflect synergistic effects among multiple metabolites, phenomena usually observed in fungal secondary metabolism. For instance, studies on the entomopathogenic fungus *Metarhizium robertsii* have demonstrated that the collective production of multiple classes of secondary metabolites enables the fungus to combat different bacteria more effectively than the metabolites produced by individual biosynthetic gene clusters alone, operating through additive and synergistic mechanisms [37].

For antifungal activity, feature F_411.325183 (putatively annotated as C_28_ oxidised sterol derivative, such as ergosterol peroxide) displayed significance (*p* = 0.04); this should be interpreted cautiously given the absence of FDR correction across multiple comparisons. However, the absence of strong correlation coefficients means that the antifungal activity cannot be linearly predictable from individual feature abundance alone. This aligns with the understanding that antifungal activity arises from multiple metabolite interactions rather than isolated compounds.

A significant negative correlation was observed between F_411.325183 (oxidised sterol-like feature) and F_375.3042615 (putatively annotated as polyunsaturated terpenoid/carotenoid-related hydrocarbon). Both metabolite classes originate from the same cellular isoprenoid pathway [38]. In fungi, this pathway branches towards sterol biosynthesis and carotenoid/terpenoid production via a phytoene synthase-dependent route [39]. This inverse production pattern is consistent with differential regulation or competition for precursors across the isoprenoid branch under the tested conditions. Carotenoids function as antioxidants involved in the quenching of reactive oxygen species (ROS) [38,39], whereas sterols are structural membrane components susceptible to oxidative modification. Oxidised sterol products have been linked to stress-responsive cellular and membrane remodelling processes [40,41]. Thus, the observed negative correlation may reflect pathway-level regulatory balancing under the tested cultivation conditions. However, it is important to note that oxidised sterol can also be produced through non-enzymatic oxidation during cultivation, extraction, or storage. Therefore, the observed relationship should be interpreted cautiously as an exploratory indication of possible branch regulation rather than definitive metabolic flux diversion.

Although Spearman correlation did not identify significant features across bioactivity endpoints, a limited number of features exhibited a trend towards association with cytotoxic activity. Feature F_717.5214 (putatively classified as a glycerophospholipid-related metabolite) and F_518.2397 (putatively annotated as Fiscalin E analogue) displayed marginal uncorrected *p*-values (*p* = 0.06) (Figure S1). While these values do not meet conventional statistical standards (*p* < 0.05), the known biological activity of fiscalin-type alkaloids in prior studies as neuroprotective [42,43] with cytotoxic properties [18,44,45] supports the probability of this candidate association. Nonetheless, given the exploratory nature of this analysis and limited sample size, findings need to be interpreted cautiously.

Importantly, no feature demonstrated consistent associations across multiple bioactivity assays. This suggests that while medium-dependent metabolites contribute to functional variation, bioactivity cannot be associated with a single feature in a simple linear manner. Instead, the data support a model in which nutrient-induced pathway regulation reshapes the metabolite profile. Also, bioactivity outcomes reflect strain-specific metabolome together with coordinated secondary metabolite expression.

#### 2.4.3. OPLS Regression Modelling for Cytotoxic Activity

To determine whether cytotoxic activity could be quantitatively explored in relation to metabolite variation across extracts, an OPLS regression model was built with cytotoxic activity as the Y variable and full LC-MS feature matrix as the X variable. Given the exploratory design of this study, this model was employed as a variable prioritisation tool rather than a predictive instrument.

**Figure 11:**
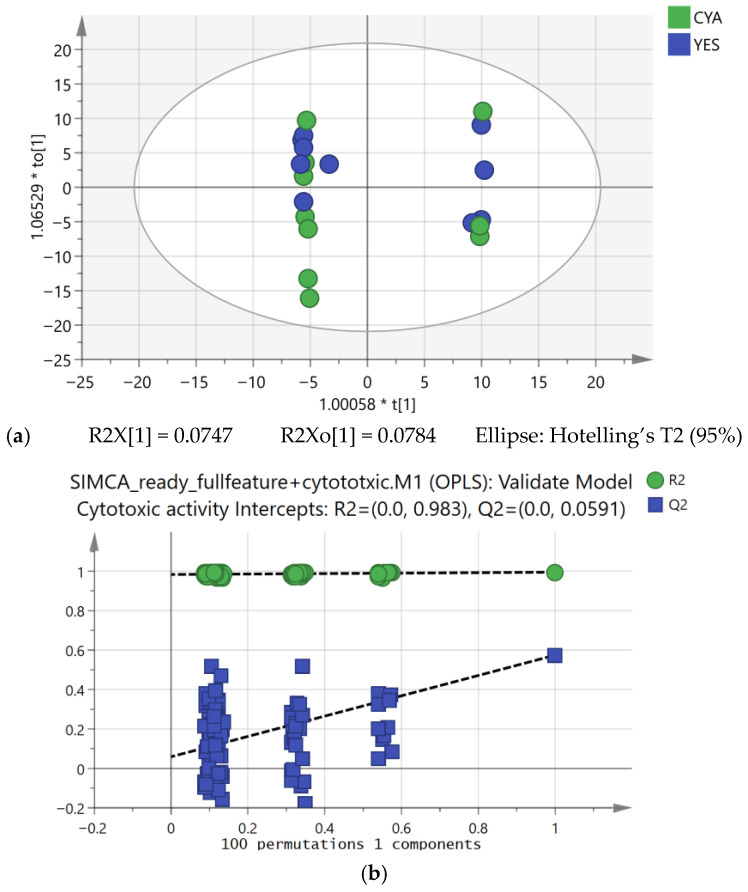
OPLS regression model constructed using cytotoxic activity as the Y-variable (axis labels reflect SIMCA 14.1 display scaling conventions, where the coefficient preceding the asterisk denotes a normalisation scalar applied to component scores for visualisation purposes). (**a**) Score plot and (**b**) permutation validation plot.

The OPLS model demonstrated high explanatory power (R^2^ = 0.983) relative to Q^2^ (0.0591), indicating that the model explains the majority of the variance within the dataset while its predictability is limited. This discrepancy could be attributed to fitting high-dimensional metabolomics data (756 features) against a small sample size (*n* = 20), where the model captures structured biological variance but lacks sufficient observations for robust cross-validation. Permutation testing (100 permutations) and CV-ANOVA (*p* < 0.05) supported that the model captures genuine structured variance within this dataset (Figure 11 and Table 2). This pattern is frequently observed in metabolomics studies with small sample sizes and high-dimensional feature matrix, where models can capture structured variance within the dataset but exhibit modest cross-validated performance [46,47]. Therefore, the regression model was interpreted mainly as a tool for variable prioritisation rather than as a predictive model for cytotoxic activity, an approach common with standard practice in metabolomics where PLS models are frequently employed to identify relevant features rather than for external prediction [48]. These results suggested cytotoxic activity to be media-associated under the studied conditions, although predictive robustness remains to be confirmed in larger replicated studies.

##### Identification of Cytotoxicity-Associated Features

Variable importance in projection (VIP) scores were used to identify features contributing most strongly to the regression model. Using a conservative threshold of VIP ≥ 1.7, sixteen features were selected as primary contributors to cytotoxic activity (Table S3).

**Figure 12:**
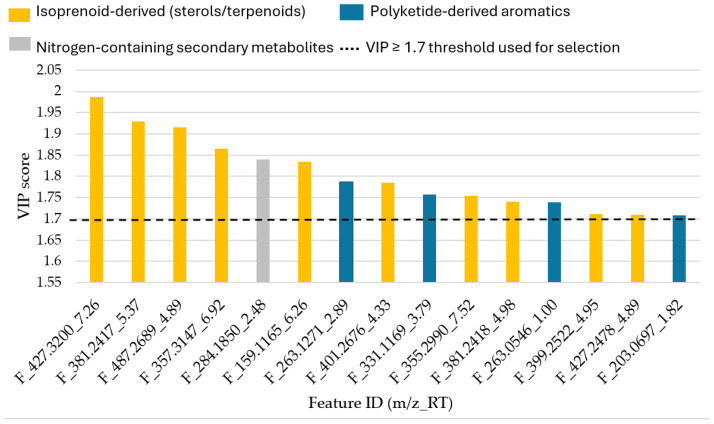
Bar plot of the VIP scores from OPLS regression model of top 15 cytotoxic-associated metabolites grouped by major biosynthetic class.

A bar plot of top VIP features (Figure 12) classified these metabolites into major biosynthetic classes. Annotation of top 15 contributors (Table S3) revealed that isoprenoid-derived sterols and terpenoids represented the dominant chemical class followed by polyketide-derived aromatic metabolites. Multiple VIP features corresponded to sterol derivatives, including oxidised sterol-like structure, prenol-lipid derivatives, and sesquiterpenoid-type metabolites.

The predominance of sterol and terpenoid derivates suggests that the activation of isoprenoid metabolism may underlie the observed cytotoxic phenotype. Sterols and polycyclic terpenoids are among the most bioactive reported classes of natural products in fungi [49,50]. Their lipophilicity and membrane affinity properties allow for integration into the mitochondrial membrane, where they can disrupt membrane potential, promote cytochrome release, and trigger intrinsic cell apoptosis [51,52,53]. In addition to membrane disrupters, several sterols and terpenoids are also known modulators of oxidative stress. Their interaction with mitochondrial electron transport components may increase ROS production, amplifying oxidative damage and inducing apoptosis [54,55]. Oxidised sterol derivates like ergosterol peroxide-like compounds have been associated with ROS-mediated cytotoxic effect in tumour models [56,57]. The structural features observed among the annotated VIP features including oxidised sterol cores, conjugated system, and hydrophobic side chains are consistent with pharmacophores commonly reported in cytotoxic natural products [5]. Importantly, no primary metabolites were identified among the top VIP contributors, reinforcing that cytotoxicity in this study is driven by specialised secondary metabolism rather than central metabolism. While direct mechanistic validation was beyond the scope of the present study, the chemical classes identified align with the established cytotoxic mechanism reported for fungal-derived sterols and terpenoids. These findings represent targeted hypotheses for future bioassay-guided investigation. Also, future work involving metabolite isolation and structural confirmation will be required to directly link specific compounds to the observed biological activities.

Comparable regression models were constructed for antibacterial and antifungal activity but did not achieve acceptable validation criteria. This contrast suggests that cytotoxic activity exhibits a more rational quantitative relationship with metabolite composition within this dataset, whereas antimicrobial responses may arise from more heterogeneous or synergistic metabolic interactions. Altogether, these findings indicate that while medium alone does not uniformly influence bioactivity patterns, the cytotoxicity activity in particular is associated with quantitative shifts in isoprenoid-related secondary metabolites in this exploratory study. Nutrient-dependent modulation of sterol and terpenoids may therefore represent a key biochemical factor underlying the observed cytotoxic responses.

### 2.5. Limitations of the Study

This study has some limitations that should be considered when interpreting the results. Due to the origin of the provided extracts, biological replicates were unavailable, limiting the assessment of intra-strain variability and potentially affecting the robustness of the multivariate model. As a result, this study is explicitly exploratory in design, and all conclusions should be regarded as hypothesis-generating rather than definitive. Although OPLS regression demonstrated high explanatory capacity for cytotoxicity, predictive performance was modest, reflecting the challenges of modelling high-dimensional metabolomics data with relatively small sample sizes. The substantial R^2^/Q^2^ gap reflects the underdetermined nature of the dataset (756 features against 20 observations) and should be interpreted as evidence of biological signal, and the model was used exclusively for variable prioritisation. Metabolite annotations were based on spectral similarity and in silico prediction (Level 2/3 confidence) and therefore require further structural confirmation. Finally, bioactivity–metabolite relationships were inferred from statistical associations rather than direct functional validation. Future studies will include biological replication, targeted quantification and isolation, and mechanistic assays to confirm relationships and refine understanding of nutrient-dependent metabolic regulation in fungi.

## 3. Materials and Methods

### 3.1. Fungal Strains and Culture Conditions

Fungal strains used in this study were obtained from the IBT Culture Collection, Department of Biotechnology and Biomedicine, Technical University of Denmark (IBT Culture Collection). The strains included multiple isolates of *Penicillium* and *Aspergillus* spp. from distinct ecological origins. Strains were identified using a polyphasic taxonomy approach, with macro- and micromorphological characterisation across six culture media (YES, CYA, OAT, PDA, MEAox, CREA) and molecular identification using established genetic markers. Species assignments were confirmed using sequence data from one or more loci, including β-tubulin, internal transcribed spacer (ITS) regions, and calmodulin, where applicable. Detailed strain identifiers and isolation sources are provided in Table 3, and detailed loci used for each strain are provided in Table S4.

The isolates were cultivated and extracted prior to this study. Extraction was performed according to the protocol described below.

The strains were cultivated on 50 mm Petri dishes on two media, CYA (yeast autolysate 5.0 g, Czapek dox broth 35.0 g, agar 20.0 g, MilliQ water 885 mL) and YES (yeast extract 20.0 g, sucrose 150.0 g, 0.5 g MgSO_4_ · 7 H_2_O, agar 20.0 g, MilliQ water 885 mL). They were incubated for ten days at 25 °C. Then, the fungus and agar were cut into pieces (approx. 5 × 5 mm) and transferred to 50 mL Falcon tubes. In total, 10 mL of ethyl acetate-isopropanol (3:1) containing 1% formic acid was added to the Falcon tubes, which were then placed in an ultrasonic bath for 60 min. The extracts were transferred to 15 mL Falcon tubes and were centrifuged (at 45,000× *g* and 4 °C for 15 min) to precipitate the spores and separate the water (residual water from the agar) in a different phase. The organic supernatant was transferred to 8 mL vials, where the extracts were dried under nitrogen flow at room temperature.

Note: Each sample was cultivated only once due to limited material availability. Thus, this study is exploratory in nature and focuses on comparative metabolomic and bioactivity patterns rather than inferential statistical testing.

### 3.2. LC-MS Acquisition and Sample Preparation

Untargeted metabolomic profiling was performed using high-performance liquid chromatography coupled with high-resolution mass spectrometry (HPLC-HRMS). A Bruker Maxis II^®^ electrospray ionisation quadrupole time-of-flight (ESI Q-TOF) instrument coupled with the Agilent 1290 Quat pump reverse phase HPLC system was used for metabolomic profiling. In total, 10 µL of sample was injected into the HPLC system with a flow rate of 1 mL/min through the Agilent C18 2.7 µm 2.1 × 100 mm column. The mobile phase consisted of solvent (A), 5% acetonitrile, and 95% water with 0.1% formic acid, and solvent (B) was 100% acetonitrile with 0.1% formic acid. A gradient elution was applied from 5% to 95% B over 10 min, followed by an isocratic hold at 95% B for 2 min. Capillary voltage of 4.5 kV, nebuliser gas at 4.5 bar, and dry temperature at 220 °C were maintained. Standardised reserpine (C_33_H_40_N_2_O_9_) was used as a calibration standard for the system. For metabolomics analysis in this study, only features detected within the first 10 min of the chromatographic run were considered, corresponding to the gradient phase. 

All LC-MS samples were prepared using methanol and Milli-Q water depending on their solubility. A final concentration of 0.3 mg/mL was used for crude fungal extracts.

### 3.3. Data Processing and Molecular Networking

Raw LC-MS data were converted to mzXML format using the Bruker software v5.1 export option and processed using MZmine v 4.8.30 [58]. The workflow included mass detection, chromatogram building, peak deconvolution, isotopic peak grouping, feature alignment, and gap filling using standard parameters. Blank subtraction was performed using the MZmine feature list blank subtraction module; features detected in blank samples (culture medium controls) were removed based on a fold change threshold of ≥5 relative to blanks (ratio type: maximum intensity comparison). Quality control (QC) samples were included to monitor analytical reproducibility throughout the dataset. However, no additional QC-based filtering was applied. Feature tables containing *m*/*z* ratio, retention time, and peak area were exported for multivariate analysis.

A molecular network was created with the Feature-Based Molecular Networking (FBMN) workflow [59] on GNPS [60]. The mass spectrometry data were first processed with MZMINE2 and the results were exported to GNPS for FBMN analysis. The data was filtered by removing all MS/MS fragment ions within +/−17 Da of the precursor *m*/*z*. MS/MS spectra were window filtered by choosing only the top 6 fragment ions in the +/−50 Da window throughout the spectrum. The precursor ion mass tolerance was set to 0.02 Da and the MS/MS fragment ion tolerance to 0.02 Da. A molecular network was then created where edges were filtered to have a cosine score above 0.7 and more than 6 matched peaks. Further, edges between two nodes were kept in the network if and only if each of the nodes appeared in each other’s respective top 10 most similar nodes. Finally, the maximum size of a molecular family was set to 100, and the lowest scoring edges were removed from molecular families until the molecular family size was below this threshold. The spectra in the network were then searched against GNPS spectral libraries [60,61]. The library spectra were filtered in the same manner as the input data. All matches kept between network spectra and library spectra were required to have a score above 0.7 and at least 6 matched peaks. The DEREPLICATOR was used to annotate MS/MS spectra [62]. The molecular networks were visualised using Cytoscape v3.10.4 software [63].

### 3.4. Feature Annotations

SIRIUS v 6.3.3 [64] was used for the annotation of top features. The parameters for the SIRIUS computation were set as default. SIRIUS and ZODIAC [65] were used for molecular formula identification, in which a zodiac score ≥ 50% was considered. CSI: Finger ID [66] was used for fingerprint prediction and CANOPUS [67,68,69] for compound class prediction where only compound class prediction with probability ≥ 60% was considered. The databases were used for structure search in SIRIUS as follows: GNPS [60], COCONUT [70], ChEMBL [71], Natural products [72,73], and PubChem [74].

Due to the exploratory nature of this study, structural annotation was focused on a subset of biologically relevant features, including the top discriminant metabolites and those associated with cytotoxic activity, rather than the full feature list.

### 3.5. Bioactivity Screening

#### 3.5.1. Cytotoxic Activity

All the fungal extracts were tested by the MTT method to evaluate their cytotoxicity against human hepatoma cell line (HepG2). ED_50_ (50% effective dose) values are summarised in Table S2. Human cancer cell lines were purchased from American Type Culture Collection (ATCC HB-8065) (ATCC, Manassas, VA, USA). Extracts were assayed in duplicate as 10-point curves with 1/200 dilution starting at 20 μg/mL. HepG2 cells were seeded at 8000 cells/well in 384-well plates (Corning 3701), (Corning Inc., Corning, NY, USA) and incubated overnight at 37 °C and 5% CO_2_. Nanolitres of the corresponding concentrations of pure compounds were added by means of a Beckman Echo 550 (Beckman Coulter, Tokyo, Japan) [75] and cells were further incubated for 72 h. MMS (methylmethanesulfonate, Sigma-Aldrich 129925, St. Louis, MO, USA), 2 mM, was used as the positive control, and DMSO 0.5% was used as the negative control. Finally, MTT dye (thiazolyl blue tetrazolium bromide, ACROS Organics 158990050, 0.5 mg/mL) (ACROS organics, Geel, Belgium) was added to each well, cells were incubated for 2–3 h, supernatant was removed, 20 μL of DMSO (100%) was added, and absorbance was measured at 570 nm. The results obtained were analysed using Genedata Screener software (Genedata Inc., Basel, Switzerland).

#### 3.5.2. Antimicrobial Activity

Antimicrobial assays were performed using a microdilution format in a sterile 96-well plate. All the fungal extracts prepared at a concentration of 6.25 mg/mL and dissolved in 100% DMSO were used for bioactivity screening. For each assay, 10 µL of extract was dispensed, followed by 90 µL of standardised microbial inoculum (final volume: 100 µL per well). Microbial inocula were standardised prior to each assay. For bacterial strains, inoculum density was determined using optical density (OD) measurements calibrated against viable counts (CFU/mL) obtained by serial dilution and plating. For filamentous fungi, inoculum density was determined by microscopic counting of conidia and adjusted to required concentration. All assays were performed in duplicate and included appropriate blanks (medium only), growth control (inoculum with vehicle-DMSO), and positive control wells.

Percentage growth inhibition was calculated as follows:

$$\%Inhibition=\left[1-\frac{\left(T_{f}\;Sample-T_{0}\;Sample\right)-\left(T_{f}\;Blank-T_{0}\;Blank\right)}{\left(T_{f}\;Growth-T_{0}\;Growth\right)-\left(T_{f}\;Blank-T_{0}\;Blank\right)}\right]\times 100$$

Absorbance was measured at 612 nm at time zero (T_0_) and after incubation (T_f_) using a microplate spectrophotometer.

##### Antibacterial Activity

Antibacterial activity was performed against *Acinetobacter baumannii* ATCC 19606, methicillin-resistant *Staphylococcus aureus* (MRSA MB5393), and *Pseudomonas aeruginosa* ATCC 5919.

Bacterial inoculum was prepared by streaking frozen stocks onto agar plates and incubating overnight at 37 °C. Single colonies were inoculated into liquid culture media and incubated overnight at 37 °C with shaking (220 rpm). Cultures were diluted in fresh medium to obtain final inoculum concentrations of approximately 5–6 × 10^5^ CFU/mL for *A. baumannii* and *P. aeruginosa*, and approximately 1.1 × 10^6^ CFU/mL for MRSA. Mueller-Hinton II (MH2) broth was used for *A. baumannii* and *P. aeruginosa*, and Brain Heart Infusion (BHI) broth for MRSA.

Aztreonam (*A. baumannii* and *P. aeruginosa*) and vancomycin (MRSA) were used as positive controls, prepared as two-fold serial dilutions. Plates were incubated statically at 37 °C for approximately 20 h prior to final measurement.

##### Antifungal Activity

Antifungal activity was assessed against *Aspergillus fumigatus* ATCC 46645, *Candida albicans* ATCC 64124, and *Candida auris* ATCC 13225 using similar microdilution approach.

Yeast inocula (*C. albicans* and *C. auris*) were prepared by culturing strains on Sabouraud Dextrose Agar (SDA) plates at 35–37 °C for 24 h. Colonies were suspended in RPMI-1640 medium buffered with HEPES and adjusted to an optical density of approximately 0.25–0.28 at 660 nm, followed by 1:100 dilution to obtain the working inoculum of 1.0 × 10^5^ CFU/mL for *C. albicans* and 1.0 × 10^6^ for *C. auris*.

Amphotericin B was used as a positive control (two-fold serial dilution). Plates were incubated statically at 37 °C for 24–30 h before final absorbance (T_f_) was recorded.

For filamentous fungi *A. fumigatus*, conidia were obtained from cultures grown on Potato Dextrose Agar (PDA). The conidial suspension was prepared in RPMI-1640 medium and adjusted to approximately 2.5 × 10^4^ CFU/mL.

Following incubation at 37 °C for 24–30 h, metabolic activity was assessed using resazurin (0.002%) as a viability indicator. Fluorescence was measured at excitation/emission wavelengths of 570/600 nm, and growth inhibition was calculated relative to controls.

### 3.6. Multivariate and Predictive Modelling

Multivariate analyses were performed in Simca v 14.0 (Umetrics, Umeå, Sweden). Principal component analysis (PCA), orthogonal partial least squares discriminant analysis (OPLS-DA), and OPLS/PLS regression were used to explore metabolome patterns. To reduce the analytical variability, data was normalised in SIMCA 14.0, and log10 transformation and Pareto scaling was applied prior to statistical analysis. Model quality was evaluated using R^2^ (explained variance) and Q^2^ (predictive ability) values, and cross-validated using permutation testing with 100 iterations, in addition to running cross-validation ANOVA (CV-ANOVA). Heatmaps, volcano plots, and corelation plots were produced using Metaboanalyst 6.0 [76].

## 4. Conclusions

This study suggests that culture medium may possibly influence the fungal metabolome at the pathway level of ten fungal strains studied here, while bioactivity outcome remains strongly strain-dependent. These findings are presented as hypothesis-generating observations arising from a pilot dataset lacking biological replication and should be interpreted within that explicit exploratory framework. Untargeted LC-HRMS metabolomics revealed medium-specific shifts in metabolite families, particularly within isoprenoid-derived sterols, terpenoids, and nitrogen-containing secondary metabolites. Although multivariate separation between YES and CYA extracts was moderate, discriminant analysis identified metabolite clusters selectively enriched in CYA nutrient conditions. Importantly, cytotoxic activity co-varied with metabolite composition, with sterol and terpenoid-related features emerging as major contributors in regression modelling. These findings are consistent with established mechanisms linking membrane-active and redox-modulating secondary metabolites to cytotoxic effects. In contrast, antibacterial and antifungal activities displayed weaker associations with individual metabolites, supporting that antimicrobial activity likely arises from strain-specific metabolite combinations. However, because biological testing was performed on crude extracts, metabolites should be regarded as putative candidate features associated with bioactivity rather than confirmed active principles. Definitive assignment of biological activity to individual metabolites would require targeted isolation, purification, and testing of pure compounds. The hypothesis-generating observations requiring future validation also include the YES-associated induction of antifungal metabolites, a putative contribution of fiscalin-type alkaloids and oxidised sterols to cytotoxicity, competitive isoprenoid branch regulation, and synergistic origins of antimicrobial activity. Future transcriptomic profiling of these strains under YES and CYA conditions would enable the direct investigation of BGCs underlying observed metabolic shifts, particularly the differentially regulated isoprenoid and nitrogen-containing metabolite pathways identified here. Collectively, this work highlights the role of nutrient-driven metabolic regulation and intrinsic biosynthetic capacity in shaping functional outcomes in fungal metabolism. By integrating cultivation strategies with statistical modelling, this study provides a systematic framework and a set of targeted hypotheses for future investigations in condition-dependent fungal chemical diversity and its implications for fungal natural product discovery.

## Figures and Tables

**Table 1:** CV-ANOVA results of the OPLS-DA model (YES vs. CYA).

Model: OPLS-DA (YES vs. CYA)	SS	DF	MS	F	*p*	SD
Total corr.	61	61	1	-	-	1
Regression	55.4309	6	9.23848	91.2378	9.03959 × 10^−27^	3.03949
Residual	5.56914	55	0.101257	-	-	0.318209

**Table 2:** CV-ANOVA results of the OPLS regression model (cytotoxicity).

Model: OPLS-Regression (Cytotoxicity)	SS	DF	MS	F	*p*	SD
Total corr.	19	19	1	-	-	1
Regression	10.9108	6	1.81847	2.92243	0.0496247	1.34851
Residual	0.08918	13	0.622245	-	-	0.788825

**Table 3:** Strain information with the details of isolation.

Species	IBT Code	Extract Code	Medium	Country	Place	Source
*Penicillium polonicum*	36874	RD3 RD6	YES CYA	Denmark	DTU Campus Lyngby	Soil
*Penicillium hordei*	36516	RD8 RD11	YES CYA	Denmark	Greenhouse	Wheat soil; Rhizosphere
*Penicillium freii*	35771	RD9 RD12	YES CYA	Denmark	Greenhouse	Soil at wheat plant; Bulk soil
*Penicillium crustosum*	36822	RD16 RD19	YES CYA	Denmark	DTU Campus Lyngby	Soil
*Penicillium velutinum*	32349	RD21 RD24	YES CYA	Malaysia	Puncak alam forest	-
*Aspergillus insuetus*	28304	RD27 RD30	YES CYA	Canada	Ottawa	Indoor air
*Aspergillus insuetus*	28267	RD34 RD37	YES CYA	Canada	Ontario	Indoor air
*Penicillium expansum*	36840	RD28 RD31	YES CYA	Denmark	DTU Campus Lyngby	Soil
*Penicillium expansum*	35795	RD35 RD38	YES CYA	Denmark	Greenhouse	Soil at wheat plant; Bulk soil
*Penicillium scabrosum*	36689	RD36 RD39	YES CYA	Denmark	Skanderborg, 8660	Forest soil

Notes: Species and strain identifiers were obtained from the IBT Culture Collection (Technical University of Denmark). Isolation source refers to the original substrate or environment from which the strain was obtained.

## Data Availability

The original contributions presented in this study are included in the article/Supplementary Materials. Further inquiries can be directed to the corresponding author(s).

## Supplementary Materials

The following supporting information can be downloaded at: https://www.mdpi.com/article/10.3390/ijms27093866/s1.

## Abbreviations

The following abbreviations are used in this manuscript:

LC-HRMS	Liquid chromatography–high-resolution mass spectrometry
PCA	Principal component analysis
OPLS-DA	Orthogonal partial least square discriminant analysis
VIP	Variable importance in projection
YES	Yeast extract sucrose medium
CYA	Czapek yeast autolysate medium
BGCs	Biosynthetic gene clusters
