# Supplementary material for: An Exploratory LC-HRMS Metabolomics Study of Culture Medium-Dependent Metabolic Variation and Bioactivity in Ten Fungal Strains
Source: Int J Mol Sci. 2026 Apr 27;27(9):3866. doi: 10.3390/ijms27093866 (PMC13163426; doi:10.3390/ijms27093866)
Supplement: Supplementary file 1 [file ijms-27-03866-s001.zip › ijms-4259291-supplementary.pdf]

Table S1. Putative structural annotations of top media-discriminant metabolic features identified by OPLS-DA, volcano plot filtering ($p < 0.05$ raw, FC ≥ 1.5), and SIRIUS/GNPS-based in silico annotation. (Annotation confidence level; Level 2 - Probable structure: GNPS spectral library match (cosine score ≥ 0.7, ≥ 6 matched peaks) or high-confidence SIRIUS/CSI:FingerID structural match with ≥ 60% posterior probability. Structure is probable but not confirmed by authentic standard; Level 2a - Reference spectrum match from GNPS library with additional formula and class confirmation. Stereochemistry and regiochemistry undetermined; Level 3 -Tentative candidate: Molecular formula confirmed (Zodiac score ≥ 63%), compound class predicted by CANOPUS (≥ 60% probability), and/or substructure match by in silico fragmentation. No reference spectrum available; Level 4 - Unknown: Unassigned feature with insufficient spectral or formula information for annotation).

Feature ID	Observed m/z	RT (min)	VIP score	FC	Adjusted p-value (FDR)	Medium enrichment	Molecular formula	Adduct	Compound class	Putative Identification	Annotation level	Annotation basis
F_395.3298_7.74	395.3298	7.74	3.87	4.48	2.22×10^{-12}	CYA	$C_{28}H_{42}O_4$	$[M+H]^+$	Ergostane steroids	Ergosterol or related sterol	3	Zodiac formula confidence 100%; 3-hydroxy Δ^5-steroid class prediction (94.1%); consistent with fungal membrane sterol biosynthesis
F_277.2156_5.54	277.2156	5.54	3.17	11.56	1.69×10^{-7}	CYA	$C_{18}H_{28}O_2$	$[M+H]^+$	Fatty acids and conjugates	Octadecadienoic acid isomer (C18:2)	3	Zodiac formula confidence 100%; lipid class prediction 93.2%; structural similarity to linoleic acid isomers; dienoic fatty acid structure.
F_716.5214_9.53	716.5214	9.53	3.73	3.39	1.99×10^{-7}	CYA	$C_{39}H_{74}NO_8P$	$[M+H]^+$	Glycerophospholipids-Phosphatidylethanolamines	PE(16:0_18:2) - diacylglycerophosphoethanolamine	2	Zodiac formula confidence 100%; MS/MS spectral match 78.9%; characteristic phospholipid fragments; isomeric ambiguity: exact regiochemistry (sn-1/sn-2) and double bond positions undetermined.
F_315.2314_6.59	315.2314	6.59	3.17	11.78	2.08×10^{-6}	CYA	$C_{21}H_{30}O_2$	$[M+H]^+$	Prenol lipids - diterpenoids	Diterpenoid carboxylic acid or ester	3	Zodiac confidence 100%; diterpenoid carboxylic acid scaffold with C21 carbon count; cyclopentane phenanthrene substructure consistent with terpenoid biosynthetic origin.
F_397.3461_8.29	397.3461	8.29	2.84	5.14	1.42×10^{-5}	CYA	$C_{28}H_{44}O$	$[M+H]^+$	Triterpenoids-ergostane steroids	Ergosterol or ergosterol isomer	2	Zodiac formula confidence 100%; MS/MS fragmentation consistent with ergosterol-class sterols; $\Delta^{5,7}$, $\Delta^{5,24}$, or $\Delta^{5,22}$ isomers cannot be distinguish without reference standards
F_599.5026_8.66	599.5026	8.66	2.89	3.46	2.94×10^{-5}	CYA	$C_{39}H_{66}O_4$	$[M+H]^+$	Fatty acid esters	Fatty acid ester dimer	3	Zodiac confidence 87.9%; consistent with hydroxy-fatty acid ester conjugate; possible 9-linoleoyloxy-

										(hydroxy-fatty acid / polyunsaturated fatty acid)		hexanoic acid or analogous structure.
F_393.3145_6.68	393.3145	6.68	2.73	2.31	8.67×10^{-5}	CYA	$C_{23}H_{46}O_2$	$[M+K]^+$	Terpenoid/ steroid alcohol	C23 hydroxylated terpenoid/fatty acid derivative	3	Zodiac confidence 63.4% ; potassium adduct ($[M+K]^+$); prenol lipid posterior 67.0%; alcohol class 82.2%; potassium adducts common in fungal extracts due to intracellular K^+ abundance.
F_409.3090_6.14	409.309	6.14	2.5	1.85	1.58×10^{-4}	CYA	$C_{28}H_{40}O_2$	$[M+H]^+$	Benzene and substituted derivates	Macrocyclic aromatic ether (tetramethyl-dioxacyclohexacosane type)	3	Zodiac confidence 100% ; macrocyclic ether with mixed aromatic and cyclic character; consistent with specialised fungal polyketide-type metabolism.
F_563.5509_6.85	563.5509	6.85	1.62	193.57	2.86×10^{-3}	CYA	$C_{36}H_{70}N_2O_2$	$[M+H]^+$	N-acyl amines - fatty acid amides	C_{36} N-acyl amide (long-chain fatty acid amide conjugate)	3	Zodiac confidence 100%; N-acyl amine posterior 86.2%; spectral match 73.9%; consistent with long-chain fatty acid conjugated to amino acid or ethanolamine; exact isomer requires MS/MS validation.
F_252.0652_1.97	252.0652	1.97	1.9	12.56	2.86×10^{-3}	CYA	$C_{15}H_9NO_3$	$[M+H]^+$	Phenolic benzenoids	Putative phenolic alkaloid	3	Zodiac confidence 79.3%; phenolic alkaloid class prediction; limited MS/MS library match available; structural confirmation required.
F_393.3145_7.16	393.3145	7.16	2.41	1.82	7.25×10^{-3}	CYA	$C_{28}H_{40}O_2$	$[M+H]^+$	Ergostane steroids	Dehydro-ergosterol derivative (tetra-unsaturated fungal sterol)	3	Zodiac confidence 100%; more unsaturated ergosterol analogue (4 fewer H = 2 extra double bonds); consistent with ergosta-5,7,9(11),22-tetraen-3β-ol or dehydroergosterol; possible pre-vitamin D_2 intermediate.
F_375.3043_7.06	375.3043	7.06	2.62	2.27	8.05×10^{-3}	CYA	$C_{38}H_{38}$	$[M+H]^+$	Prenol lipids - carotenoid-related	Polyunsaturated terpenoid hydrocarbon	3	Zodiac confidence 100%; C_{28} polyunsaturated terpenoid hydrocarbon; isoprenoid branching pattern consistent with degraded carotenoid fragment or triterpenoid hydrocarbon; negatively correlated with F_411.3252 (see note).

F_322.1293_3.44	322.1293	3.44	3.01	36.91	8.50×10^{-3}	CYA	$C_{17}H_{15}N_5O_2$	$[M+H]^+$	Indole alkaloids-tryptophan derivatives	Tryhistatin or structural analog	2	Zodiac confidence 100%; perfect formula match to known fungal indole alkaloid; characteristic indole MS/MS fragmentation pattern
F_411.3252_7.50	411.3252	7.5	3.21	5.16	8.53×10^{-3}	CYA	$C_{28}H_{42}O_2$	$[M+H]^+$	Steroids and steroids derivatives	C_{28} oxidized sterol (dihydroxy or keto-hydroxy derivative)	3	Zodiac confidence 100% ; two oxygens suggest diol, hydroxy-ketone, or peroxide functionality; consistent with oxidised ergosterol derivative (e.g. ergosterol peroxide or hydroxy-ergosterol); negatively correlated with F_375.3043 (isoprenoid branch competition hypothesis).
F_726.5512_7.26	726.5512	7.26	1.78	5.5	1.41×10^{-2}	CYA	$C_{41}H_{75}NO_8$	$[M+O+H]^+$	Glycosphingolipids	HexCer(d19:2_16:1) - hexosylceramide	2a	100% Zodiac and SIRIUS confidence; MS/MS matches HexCer fragmentation pattern; explains 41/41 peaks (86.9% intensity); sugar identity (glucose vs galactose), double bond positions, and stereochemistry undetermined.
F_279.2309_6.45	279.2309	6.45	1.41	5.31	2.30×10^{-2}	CYA	$C_{18}H_{30}O_2$	$[M+H]^+$	Fatty acyl / prenol lipid (ambiguous)	Linoleic acid or diterpenoid derivative	3	Zodiac confidence 100% ; conflicting database matches; formula confirmed as $C_{18}H_{30}O_2$; consistent with terpenoid-like prenol lipid or unsaturated C18 fatty acid derivative; novel derivative or poorly represented in current databases.
F_198.1125_1.00	198.1125	1	1.54	4.79	2.30×10^{-2}	CYA	$C_{10}H_{15}NO_3$	$[M+H]^+$	Amino acids & derivatives	Amino acid derivative	3	Zodiac confidence 97.5%; amino acid derivative class prediction; limited structural specificity from available MS/MS data; structural confirmation required.
F_445.3153_5.37	445.3153	5.37	1.5	4.6	2.30×10^{-2}	CYA	$C_{24}H_{44}O_7$	$[M+H]^+$	Terpenoids - diterpenoids	Terracyclic diterpenoid derivative	3	Zodiac confidence 85.1%; tetracyclic diterpenoid glycoside annotation; high oxygen content (7 oxygens) consistent with glycosylated diterpenoid; glycosylated diterpenoid structures common in fungal secondary metabolism.
F_429.3359_7.19	429.3359	7.19	2.14	3.2	2.30×10^{-2}	CYA	$C_{28}H_{44}O_3$	$[M+H]^+$	Ergostane steroids	Hydroxy seco-steroid	3	Zodiac confidence 100% ; vitamin D_3 analogue substructure; consistent with ergosterol conversion to seco-steroid via photolysis or oxidative ring-opening; cis/trans

												stereochemistry unresolved.
F_760.5116_8.15	760.5116	8.15	2.18	2.94	2.30 × 10^{-2}	CYA	$C_{40}H_{74}NO_{10}P$	$[M+H]^+$	Glycerophospholipids	PS(18:2_16:0) - phosphatidylserine	2a	Zodiac confidence 100%; phosphatidylserine posterior probability 99.5%; characteristic serine head group fragments; fatty acid regiochemistry (sn-1 vs sn-2) and double bond positions undetermined.
F_316.2114_0.91	316.2114	0.91	1.45	2.36	2.30 × 10^{-2}	CYA	$C_{12}H_{25}N_7O_3$	$[M+H]^+$	Alpha amino acids and derivatives	Modified peptide/amino acid derivative	3	Zodiac confidence 68.0%; heptazole (7-nitrogen) compound suggestive of modified peptide or nitrogen-rich amino acid conjugate; limited structural specificity.
F_702.5065_8.70	702.5065	8.7	1.86	2.35	2.36 × 10^{-2}	CYA	$C_{38}H_{72}NO_8P$	$[M+H]^+$	Glycerophospholipids	PE (18:2/15:0) - phosphatidylethanolamine	2a	Zodiac confidence 100%; MS/MS matches PE fragmentation; Tanimoto similarity 74.6–79.5%; characteristic ethanolamine head group fragments; contains rare 15:0 (odd-chain) fatty acid; sn-1/sn-2 regiochemistry undetermined.
F_279.2317_6.24	279.2317	6.24	1.64	8.23	2.63 × 10^{-2}	CYA	$C_{18}H_{30}O_2$	$[M+H]^+$	Fatty acids and conjugates	Linoleic acid or its derivatives (octadecatrienoic acid)	3	Zodiac confidence 100%; (2E,9E,11E)-octadeca-2,9,11-trienoic acid as substructure; consistent with C18 polyunsaturated fatty acid metabolism.
F_427.3200_7.07	427.32	7.07	2.45	2.12	2.63 × 10^{-2}	CYA	$C_{28}H_{42}O_3$	$[M+H]^+$	Steroids and steroids derivatives	Polyoxygenated seco-steroid derivative (C_{28} modified steroid)	3	Zodiac confidence 100%; multiple hydroxyl groups, possible epoxide rings; highly modified fungal natural product consistent with polyoxygenated C_{28} terpenoid/steroid; potential bioactive component.
F_518.2397_3.63	518.2397	3.63	2.55	71.14	3.70 × 10^{-2}	CYA	$C_{28}H_{31}N_5O_5$	$[M+H]^+$	Quinazoline alkaloids	Fiscalin E or structural analog	2	Zodiac confidence 98.4%; structural similarity 61.2%; putatively annotated as Fiscalin E, a known Penicillium quinazoline alkaloid; cytotoxic and antimicrobial properties reported in literature.
F_443.3151_6.80	443.3151	6.8	2.2	5.12	3.86 × 10^{-2}	CYA	$C_{28}H_{42}O_4$	$[M+H]^+$	Steroids and steroids derivatives	Steroid ester (oxidised steroid)	3	Zodiac confidence 100%; steroid ester annotation consistent with oxidised steroid conjugate; ergostane core (1OH), ester linkage (2O), extra hydroxyl or keto modification (1O). DBE= 8 (ergostane DBE 7 + ester carbonyl DBE 1).

												Confirm distinction of ester vs peroxide requires MS/MS neutral loss or NMR.

*All annotations are putative. Definitive structural confirmation requires isolation and NMR characterisation or comparison with authentic reference standards. All features are enriched in CYA medium relative to YES medium. FC = fold change (CYA/YES). p(FDR-adjusted) = adjusted p-value using Benjamini-Hochberg false discovery rate correction. Zodiac (%) = molecular formula confidence score.

Table S2. Z-score normalised bioactivity profile of fungal extracts across cytotoxic, antibacterial and antifungal activities.

**Sample name**	**Cytotoxic activity**	***A.fumigatus* inhibition**	***C. albicans* inhibition**	***C. auris* inhibition**	**Z-score antifungal activity**	***A.baumannii* inhibition**	***P.aeruginosa* inhibition**	***S.aureus* inhibition**	**Z-score antibacterial activity**
RD11_CYA_1	0	0	0	0	-0.57	0	70.6	0	1.15
RD11_CYA_2	0	0	0	0	-0.57	0	70.6	0	1.15
RD11_CYA_3	0	0	0	0	-0.57	0	70.6	0	1.15
RD12_CYA_1	0	0	0	0	-0.57	0	0	61.4	0.18
RD12_CYA_2	0	0	0	0	-0.57	0	0	61.4	0.18
RD12_CYA_3	0	0	0	0	-0.57	0	0	61.4	0.18
RD16_YES_1	0	0	0	0	-0.57	0	0	0	-0.37
RD16_YES_2	0	0	0	0	-0.57	0	0	0	-0.37
RD16_YES_3	0	0	0	0	-0.57	0	0	0	-0.37
RD19_CYA_1	0	0	68.5	0	0.035	0	0	0	-0.37
RD19_CYA_2	0	0	68.5	0	0.03	0	0	0	-0.37
RD19_CYA_3	0	0	68.5	0	0.03	0	0	0	-0.37
RD21_YES_1	0	0	0	0	-0.57	0	0	0	-0.37
RD21_YES_2	0	0	0	0	-0.57	0	0	0	-0.37
RD21_YES_3	0	0	0	0	-0.57	0	0	0	-0.37
RD24_CYA_1	0	0	93.6	88.1	1.19	0	0	0	-0.37
RD24_CYA_2	0	0	93.6	88.1	1.19	0	0	0	-0.37
RD24_CYA_3	0	0	93.6	88.1	1.19	0	0	0	-0.37
RD27_YES_1	98	48.2	0	0	-0.003	0	0	98.8	0.52
RD27_YES_2	97	48	0	0	-0.005	0	0	98.8	0.52
RD27_YES_3	98	47	0	0	-0.005	0	0	98.8	0.52
RD28_YES_1	99	79	94.8	98.7	2.25	96.7	0	0	0.89
RD28_YES_2	99	79	94.8	98.8	2.25	96.7	0	0	0.89
RD28_YES_3	99	79	94.8	98.8	2.25	96.7	0	0	0.89
RD30_CYA_1	99	0	0	0	-0.57	0	0	100	0.53
RD30_CYA_2	98	0	0	0	-0.57	0	0	100	0.53
RD30_CYA_3	96	0	0	0	-0.57	0	0	100	0.53
RD31_CYA_1	100	46.2	0	0	-0.03	52.4	0	0	0.31
RD31_CYA_2	100	46.2	0	0	-0.03	52.4	0	0	0.31
RD31_CYA_3	100	46.2	0	0	-0.03	52.4	0	0	0.31
RD34_YES_1	100	56	28.1	0	0.34	0	0	100	0.53

RD34_YES_2	100	56	28.1	0	0.34	0	0	100	0.53
RD34_YES_3	100	55	27.5	0	0.33	0	0	100	0.53
RD35_YES_1	95	70.7	92.5	72.9	1.86	53.1	0	0	0.32
RD35_YES_2	93	70.7	92.5	72.9	1.86	53.1	0	0	0.32
RD35_YES_3	93	70.7	92.5	73	1.86	53.1	0	0	0.32
RD36_YES_1	0	0	0	0	-0.57	0	0	0	-0.37
RD36_YES_2	0	0	0	0	-0.57	0	0	0	-0.37
RD36_YES_3	0	0	0	0	-0.57	0	0	0	-0.37
RD37_CYA_1	0	0	0	0	-0.57	0	0	0	-0.37
RD37_CYA_2	0	0	0	0	-0.57	0	0	0	-0.37
RD37_CYA_3	0	0	0	0	-0.57	0	0	0	-0.37
RD38_CYA_1	100	56.6	0	0	0.09	0	0	0	-0.37
RD38_CYA_2	100	56.6	0	0	0.09	0	0	0	-0.37
RD38_CYA_3	100	56.6	0	0	0.09	0	0	0	-0.37
RD39_CYA_1	0	0	78.5	0	0.12	0	0	0	-0.37
RD39_CYA_2	0	0	78.5	0	0.12	0	0	0	-0.37
RD39_CYA_3	0	0	78.5	0	0.12	0	0	0	-0.37
RD3_YES_1	0	0	0	0	-0.57	0	0	0	-0.37
RD3_YES_2	0	0	0	0	-0.57	0	0	0	-0.37
RD3_YES_3	0	0	0	0	-0.57	0	0	0	-0.37
RD6_CYA_1	0	0	0	0	-0.57	0	0	0	-0.37
RD6_CYA_2	0	0	0	0	-0.57	0	0	0	-0.37
RD6_CYA_3	0	0	0	0	-0.57	0	0	0	-0.37
RD8_YES_1	0	0	0	0	-0.57	0	0	0	-0.37
RD8_YES_2	0	0	0	0	-0.57	0	0	0	-0.37
RD8_YES_3	0	0	0	0	-0.57	0	0	0	-0.37
RD9_YES_1	0	0	51.1	0	-0.12	0	0	0	-0.37
RD9_YES_2	0	0	51.1	0	-0.12	0	0	0	-0.37
RD9_YES_3	0	0	51.1	0	-0.12	0	0	0	-0.37

* Sample names are formatted as: strain code_culture medium_technical replicate number. For example, RD11_CYA_1 refers to strain RD11 grown on CYA medium and analysed as technical replicate 1. The replicate numbers refer to repeated analysis of same extract rather than individual biological replicates. The correspondence between RD strain code and fungal species is provided in Table 3 of the main manuscript.

* Inhibiton refers % inhibition (average of duplicates); where positive control is 100. Z-score calculation: for each extract activity,

$$z = \frac{x - \mu}{\sigma}$$

Where x is raw percentage inhibition of extract, μ is mean percentage inhibition across all extracts for organism and σ is the corresponding standard deviation. Positive Z score (Z>1) indicate above average inhibitory activity relative to th extract panel; negative Z-score indicate below average activity. Extracts showing no measurable inhibition at tested concentration were assigned raw value of zero prior to normalisation.

Figure S1. Spearman rank corelation p-value matrix between media-discriminant metabolite features and measured bioactivity endpoints.

	395.3298	393.3145	443.3151	411.3252	760.5116	445.3153	252.0652	198.1125	563.5509	315.2314	393.3145	429.3359	397.3461
395.3298	NA	0.000125	0.012315	0.39542	0.83323	0.20417	0.49625	0.82796	0.95966	0.20423	0.56965	0.8636	0.1168
393.3145	0.000125	NA	0.006325	0.08547	0.96817	0.39148	0.81803	0.93406	0.44425	0.14042	0.23496	0.61967	0.62458
443.3151	0.012315	0.006325	NA	0.40549	0.6259	0.014518	0.68192	0.78157	0.28625	0.36788	0.59909	0.19897	0.26106
411.3252	0.39542	0.08547	0.40549	NA	0.60234	0.96699	0.7436	0.94227	0.72619	1	0.115	0.67938	0.62444
760.5116	0.83323	0.96817	0.6259	0.60234	NA	0.15667	0.010296	0.081708	0.5383	0.57576	0.75697	0.5819	0.39042
445.3153	0.20417	0.39148	0.014518	0.96699	0.15667	NA	0.010431	0.28199	0.37979	0.11705	0.71496	0.62147	0.37873
252.0652	0.49625	0.81803	0.68192	0.7436	0.010296	0.010431	NA	3.45E-06	0.13306	0.079563	0.21405	0.63122	0.079683
198.1125	0.82796	0.93406	0.78157	0.94227	0.081708	0.28199	3.45E-06	NA	0.034726	0.25158	0.12103	0.28637	0.25659
563.5509	0.95966	0.44425	0.28625	0.72619	0.5383	0.37979	0.13306	0.034726	NA	0.59052	0.04103	0.28446	0.42619
315.2314	0.20423	0.14042	0.36788	1	0.57576	0.11705	0.079563	0.25158	0.59052	NA	0.75426	0.20285	0.28008
393.3145	0.56965	0.23496	0.59909	0.115	0.75697	0.71496	0.21405	0.12103	0.04103	0.75426	NA	0.38609	0.4093
429.3359	0.8636	0.61967	0.19897	0.67938	0.5819	0.62147	0.63122	0.28637	0.28446	0.20285	0.38609	NA	0.12512
397.3461	0.1168	0.62458	0.26106	0.62444	0.39042	0.37873	0.079683	0.25659	0.42619	0.28008	0.4093	0.12512	NA
375.3043	0.20786	0.035368	0.083049	0.000431	0.90688	0.093812	0.72156	0.8939	0.57624	0.75252	0.49403	0.89024	0.18807
Anti-bacte	0.88166	0.75459	0.38062	0.90748	0.089976	0.57768	0.38104	0.52725	0.58856	0.34188	0.16633	0.40327	0.46387
Cytotoxic	0.87675	0.96381	0.71184	0.85287	0.45869	0.20065	0.12539	0.20065	0.78286	0.78899	0.15881	0.8308	0.28034
Anti-fungal	0.83395	0.8152	0.54163	0.041765	0.62606	0.62534	0.78751	0.53901	0.52321	0.56618	0.085116	0.31547	0.68681
518.2397	0.89303	0.25072	0.40129	0.7718	0.14774	0.46029	0.81105	0.37392	0.06617	0.74872	0.036771	0.49976	0.34625
322.1293	0.36919	0.1546	0.62115	0.66771	0.41871	0.92543	0.47437	0.15201	0.065459	0.49172	0.21141	0.43061	0.56413
316.2114	0.86853	0.55357	0.60897	0.42141	0.075068	0.20555	0.32909	0.28199	0.71145	0.38004	0.14096	0.38788	0.15424
427.32	0.48918	0.60263	0.52931	0.66926	0.038123	0.32093	0.089537	0.28126	0.2983	0.6085	0.41325	0.098153	0.18112
409.309	0.31911	0.38544	0.44019	0.47371	0.036865	0.31004	0.05422	0.17944	0.34733	0.084477	0.48117	0.6617	0.15021
716.5214	0.12897	0.16822	0.66398	0.88204	0.067816	0.009356	0.10849	0.35561	0.63259	0.20182	0.40863	0.88508	0.39273
599.5026	0.5137	0.53878	0.60249	0.69465	0.014774	0.0145	0.037041	0.19906	0.32472	0.49296	0.17033	0.54293	0.091741
277.2156	0.1449	0.32841	0.25574	0.63102	0.005605	0.001232	0.015189	0.13091	0.21164	0.45665	0.11123	0.80226	0.31532
702.5065	0.89906	0.72982	0.84508	0.66771	0.036859	0.74999	0.36007	0.123	0.076424	0.34263	0.12863	0.3731	0.54721
726.5512	0.65047	0.57186	0.90535	0.87395	0.026341	0.6478	0.25113	0.19455	0.085218	0.83517	0.19515	0.48189	0.39651

	375.3043	Anti-bacte	Cytotoxic	Anti-fungal	518.2397	322.1293	316.2114	427.32	409.309	716.5214	599.5026	277.2156	702.5065	726.5512
395.3298	0.20786	0.88166	0.87675	0.83395	0.89303	0.36919	0.86853	0.48918	0.31911	0.12897	0.5137	0.1449	0.89906	0.65047
393.3145	0.035368	0.75459	0.96381	0.8152	0.25072	0.1546	0.55357	0.60263	0.38544	0.16822	0.53878	0.32841	0.72982	0.57186
443.3151	0.083049	0.38062	0.71184	0.54163	0.40129	0.62115	0.60897	0.52931	0.44019	0.66398	0.60249	0.25574	0.84508	0.90535
411.3252	0.000431	0.90748	0.85287	0.041765	0.7718	0.66771	0.42141	0.66926	0.47371	0.88204	0.69465	0.63102	0.66771	0.87395
760.5116	0.90688	0.089976	0.45869	0.62606	0.14774	0.41871	0.075068	0.038123	0.036865	0.067816	0.014774	0.005605	0.036859	0.026341
445.3153	0.093812	0.57768	0.20065	0.62534	0.46029	0.92543	0.20555	0.32093	0.31004	0.009356	0.0145	0.001232	0.74999	0.6478
252.0652	0.72156	0.38104	0.12539	0.78751	0.81105	0.47437	0.32909	0.089537	0.05422	0.10849	0.037041	0.015189	0.36007	0.25113
198.1125	0.8939	0.52725	0.20065	0.53901	0.37392	0.15201	0.28199	0.28126	0.17944	0.35561	0.19906	0.13091	0.123	0.19455
563.5509	0.57624	0.58856	0.78286	0.52321	0.06617	0.065459	0.71145	0.2983	0.34733	0.63259	0.32472	0.21164	0.076424	0.085218
315.2314	0.75252	0.34188	0.78899	0.56618	0.74872	0.49172	0.38004	0.6085	0.084477	0.20182	0.49296	0.45665	0.34263	0.83517
393.3145	0.49403	0.16633	0.15881	0.085116	0.036771	0.21141	0.14096	0.41325	0.48117	0.40863	0.17033	0.11123	0.12863	0.19515
429.3359	0.89024	0.40327	0.8308	0.31547	0.49976	0.43061	0.38788	0.098153	0.6617	0.88508	0.54293	0.80226	0.3731	0.48189
397.3461	0.18807	0.46387	0.28034	0.68681	0.34625	0.56413	0.15424	0.18112	0.15021	0.39273	0.091741	0.31532	0.54721	0.39651
375.3043	NA	0.97702	0.80432	0.31713	0.54987	0.57502	0.75798	0.59243	0.74975	0.095878	0.048635	0.080261	0.5723	0.67907
Anti-bacte	0.97702	NA	0.005088	0.27105	0.66739	0.99476	0.82797	0.25482	0.18922	0.11743	0.29519	0.31439	0.72723	0.86604
Cytotoxic	0.80432	0.005088	NA	0.014984	0.066428	0.86947	0.13031	0.13749	0.16713	0.061951	0.13749	0.41812	0.86947	0.95633
Anti-fungal	0.31713	0.27105	0.014984	NA	0.30735	0.69469	0.96981	0.3725	0.86024	0.70817	0.40514	0.39036	0.45332	0.19859
518.2397	0.54987	0.66739	0.066428	0.30735	NA	0.002781	0.005237	0.41923	0.54265	0.72575	0.49278	0.22611	0.029769	0.2136
322.1293	0.57502	0.99476	0.86947	0.69469	0.002781	NA	0.019057	0.40851	0.54441	0.66858	0.33084	0.26679	0.003705	0.089404
316.2114	0.75798	0.82797	0.13031	0.96981	0.005237	0.019057	NA	0.28126	0.14424	0.32218	0.17568	0.047888	0.016032	0.003324
427.32	0.59243	0.25482	0.13749	0.3725	0.41923	0.40851	0.28126	NA	0.00314	0.008663	0.00013	0.021589	0.007296	0.018242
409.309	0.74975	0.18922	0.16713	0.86024	0.54265	0.54441	0.14424	0.00314	NA	1.98E-05	0.000409	0.020053	0.020415	0.005236
716.5214	0.095878	0.11743	0.061951	0.70817	0.72575	0.66858	0.32218	0.008663	1.98E-05	NA	3.11E-07	3.37E-05	0.023646	0.027294
599.5026	0.048635	0.29519	0.13749	0.40514	0.49278	0.33084	0.17568	0.00013	0.000409	3.11E-07	NA	4.98E-06	0.002914	0.003898
277.2156	0.080261	0.31439	0.41812	0.39036	0.22611	0.26679	0.047888	0.021589	0.020053	3.37E-05	4.98E-06	NA	0.010216	0.002565
702.5065	0.5723	0.72723	0.86947	0.45332	0.029769	0.003705	0.016032	0.007296	0.020415	0.023646	0.002914	0.010216	NA	1.94E-05
726.5512	0.67907	0.86604	0.95633	0.19859	0.2136	0.089404	0.003324	0.018242	0.005236	0.027294	0.003898	0.002565	1.94E-05	NA

Table S3. Top variable importance in projection (VIP) features from OPLS regression modelling of cytotoxic activity (VIP ≥ 1.70), with putative structural annotations. (Annotation confidence level; Level 2 -Probable structure: high-confidence SIRIUS/CSI:FingerID structural match with ≥ 60% posterior probability, near-complete spectral explanation (> 95% of peaks), and/or Tanimoto structural similarity > 60%. Structure is probable but not confirmed by authentic standard; Level 3-Tentative candidate: molecular formula confirmed (Zodiac score ≥ 63%), compound class predicted by CANOPUS (≥ 60% probability). No reference spectrum available at Level 2 confidence; substructure or class match only).

**Feature ID**	**Observed m/z**	**RT (min)**	**VIP score**	**Molecular formula**	**Adduct**	**Broad compound class**	**Putative Identification**	**Annotation level**	**Annotation basis**
F_427.3200_7.26	427.32	7.26	1.99	$C_{28}H_{42}O_3$	$[M+H]^+$	Steroids & steroid derivatives	Polyoxygenated C_{28} seco-steroid derivative (multi-hydroxylated; possible epoxide/oxetane rings)	3	Zodiac formula confidence 100%. Multiple hydroxyl and epoxide functionalities inferred from molecular formula and fragmentation. Highly modified fungal natural product; consistent with oxidative ergostane ring-opening products known from Penicillium and Aspergillus spp.
F_381.2417_5.37	381.2417	5.37	1.92	$C_{23}H_{34}O_3$	$[M+Na]^+$	Terpenoids - diterpenoids	C_{23} diterpenoid or truncated sterol derivative	3	Zodiac formula confidence 100%. Sodium adduct consistent with terpenoid alcohol or ester; $C_{23}H_{34}O_3$ formula consistent with a nor-steroid or diterpenoid

									carboxylic acid scaffold. Structural specificity limited by absence of reference spectrum.
F_487.2689_4.89	487.2689	4.89	1.91	$C_{26}H_{40}O_7$	$[M+Na]^+$	Meroterpenoids	Andrastin-type meroterpenoid or structural analog (C_{26} meroterpenoid; Deoxocitreoheptyl or related)	3	Zodiac formula confidence 65.0% (moderate); oxygenation pattern (7 oxygens) consistent with Andrastin-family meroterpenoids previously isolated from Penicillium spp. Moderate confidence due to partial formula assignment.
F_357.3147_6.92	357.3147	6.92	1.86	$C_{25}H_{40}O$	$[M+H]^+$	Steroids - Δ^5-sterols	C_{25} diene sterol, 3β-hydroxy-Δ^5-sterol; likely cholestadienol or ergostadienol isomer	3	Zodiac formula confidence 100%; CANOPUS classification consistent with 3β-hydroxy-Δ^5-sterol (very high confidence). $C_{25}H_{40}O$ formula with single oxygen and degree of unsaturation consistent with a diene sterol. Exact isomer (cholestadienol vs ergostadienol) cannot be assigned without reference standard.
F_284.1850_2.48	284.185	2.48	1.84	$C_{15}H_{25}NO_4$	$[M+H]^+$	Carboxylic acids and derivatives	C_{15} nitrogen-containing carboxylic acid or amino acid conjugate	3	Zodiac formula confidence 99.9%. Nitrogen atom and four oxygens consistent with amino acid conjugate or N-acyl carboxylic acid derivative. Limited MS/MS library match; class-level annotation only.
F_159.1165_6.26	159.1165	6.26	1.83	$C_{12}H_{14}$	$[M+H]^+$	Terpenoids - sesquiterpenoids	Guaiane-type sesquiterpenoid core; trimethylindene or related polycyclic aromatic hydrocarbon	3	Zodiac formula confidence 100%. $C_{12}H_{14}$ formula with high degree of unsaturation (7) consistent with polycyclic aromatic hydrocarbon representing the core of a guaiane-type sesquiterpenoid or a fragment ion of a larger sesquiterpenoid. Guaiane skeleton documented in Aspergillus and Penicillium spp. with reported cytotoxic and anti-inflammatory properties.
F_263.1271_2.89	263.1271	2.89	1.79	$C_{15}H_{18}O_4$	$[M+H]^+$	Benzenoids - phenyl ethers	Dimethoxychromene derivative; related to 1-(5,8-dimethoxy-2,2-dimethyl-2H-chromene-6-yl)ethan-1-one (anisole-type)	3	Zodiac formula confidence 100%. MS/MS fragmentation consistent with methoxylated benzopyran scaffold; structural similarity to dimethoxychromene natural products reported from fungal polyketide biosynthesis.
F_401.2676_4.33	401.2676	4.33	1.78	$C_{25}H_{36}O_4$	$[M+H]^+$	Steroids - oxosteroid esters	17-acetyloxy steroid acetate or closely related steroid diester	3	Zodiac and SIRIUS formula confidence 100%. CANOPUS classification: steroids 67.6%, esters 67.6%. Structural similarity > 54% Tanimoto. Two ester groups inferred from molecular formula; consistent with 17α-acetyloxy substituted steroid framework

									characteristic of modified fungal or marine steroids.
F_331.1169_3.79	331.1169	3.79	1.76	$C_{18}H_{18}O_6$	$[M+H]^+$	Benzenoids - benzopyranoid lactones	Polyoxygenated benzopyranoid lactone; polymethoxycoumarin or biphenyl ether lactone	3	100% Zodiac, 99.96% SIRIUS formula confidence. CANOPUS classification: benzenoids 90.4%, lactones 65.6%, alkyl aryl ethers 63.8%. Six oxygens and high degree of unsaturation consistent with polyoxygenated fungal polyketide; moderate database similarity suggests possible novel or underrepresented compound within this structural family.
F_355.2990_7.52	355.299	7.52	1.75	$C_{25}H_{38}O$	$[M+H]^+$	Prenol lipids - diterpenoids	C_{25} prenol lipid; likely diterpenoid alcohol or sesterterpene derivative	3	Zodiac formula confidence 100%. Single oxygen with C_{25} carbon skeleton consistent with diterpenoid alcohol or sesterterpene; isoprenoid branching pattern inferred from degree of unsaturation and CANOPUS lipid class classification.
F_381.2418_4.98	381.2418	4.98	1.74	$C_{23}H_{34}O_3$	$[M+Na]^+$	Steroids - 3-oxosteroids	16α-ethyl-3-oxosteroid or closely related pregnane derivative	2	Zodiac and SIRIUS formula confidence 100% ; 57/57 MS/MS peaks explained (100%); Tanimoto structural similarity 65.7%; CANOPUS: steroids 95.6%, 3-oxosteroids 94.4%. High-confidence assignment approaching Level 2 identification. Characteristic pregnane steroid framework.
F_263.0546_1.00	263.0546	1	1.74	$C_{13}H_{10}O_6$	$[M+H]^+$	Benzenoids - hydroxybenzenoids	1-hydroxy-2-unsubstituted polyoxygenated benzenoid	3	Zodiac formula confidence 100%. Six oxygens and aromatic ring system consistent with polyhydroxylated benzenoid or phenolic lactone. Class-level assignment by CANOPUS; compound-level identity requires further structural elucidation.
F_399.2522_4.95	399.2522	4.95	1.71	$C_{25}H_{34}O_4$	$[M+H]^+$	Steroids - pregnane steroids	17α-(acetyloxy)-6,16α-dimethylpregna-4,6-diene-3,20-dione or closely related analog	2	Zodiac and SIRIUS formula confidence 100%; 98.5% of MS/MS peaks explained; Tanimoto structural similarity 67–70%; CANOPUS: gluco/mineralocorticoid/progestogen derivative. Very high confidence assignment; characteristic semi-synthetic progestin or modified marine steroid scaffold.
F_427.2478_4.89	427.2478	4.89	1.71	$C_{24}H_{36}O_5$	$[M+Na]^+$	Prenol lipids - diterpenoids	C_{24} prenol lipid; oxygenated diterpenoid derivative	3	Zodiac formula confidence 90.5%; five oxygens with C_{24} carbon skeleton consistent with highly oxygenated diterpenoid or terpenoid ester. Structural specificity limited; class-level annotation by CANOPUS lipid classification.

F_203.0697_1.82	203.0697	1.82	1.71	$C_{12}H_{10}O_3$	$[M+H]^+$	Benzenoids - simple coumarins	8-ethenyl-7-hydroxycoumarin or benzoxazinone analog	3	Zodiac formula confidence 100%; CANOPUS: simple coumarins, 1-hydroxy-2-unsubstituted benzenoids (55%); Tanimoto similarity 68–69%. Coumarin framework with alkene substitution well-supported; exact regiochemistry (8-ethenyl vs allyloxy substitution) cannot be assigned without authentic standard.

*All annotations are putative. Definitive structural confirmation requires isolation and NMR characterisation or comparison with authentic reference standards.

Table S4. Molecular loci used for the identification of fungal strains from the IBT culture collection

**Species**	**IBT code**	**Analysed loci**
Penicillium polonicum	36874	ITS
Penicillium hordei	36516	β-tubulin
Penicillium freii	35771	Not specified
Penicillium crustosum	36822	β-tubulin, calmodulin, ITS
Penicillium velutinum	32349	β-tubulin
Aspergillus insuetus	28304	β-tubulin
Aspergillus insuetus	28267	β-tubulin
Penicillium expansum	36840	β-tubulin, ITS
Penicillium expansum	35795	β-tubulin, ITS
Penicillium scabrosum	36689	β-tubulin

*Information for *Penicillium freii* was not available at the time of manuscript preparation; however identification was performed and verified by IBT culture collection using standard polyphasic taxonomy.
